# Heterogeneity of the adult mammalian forebrain neurogenic ependyma: A comprehensive cellular map

**DOI:** 10.4103/NRR.NRR-D-24-00789

**Published:** 2025-04-29

**Authors:** Xuejiao Yang, Yuchen Mu, Qianxiang Wu, Liqiang Zhou, Orion R. Fan, Quan Lin, Wenmin Zhu, Yi Eve Sun

**Affiliations:** 1 Shanghai Institute of Stem Cell Research and Clinical Translation, East Hospital, Tongji University School of Medicine, Shanghai, China; 2 Stem Cell Translational Research Center, Tongji Hospital, Tongji University School of Medicine, Shanghai, China; 3 Department of Psychiatry and Biobehavioral Sciences, David Geffen School of Medicine, University of California, Los Angeles, Los Angeles, CA, USA

**Keywords:** endothelial-like cells, ependymal cells, heterogeneity, immunocytochemical analyses, lateral ventricles, neural repair, neural stem cells, neurogenic, pericyte-like cells, single-cell RNA sequencing

## Abstract

**Figure:**
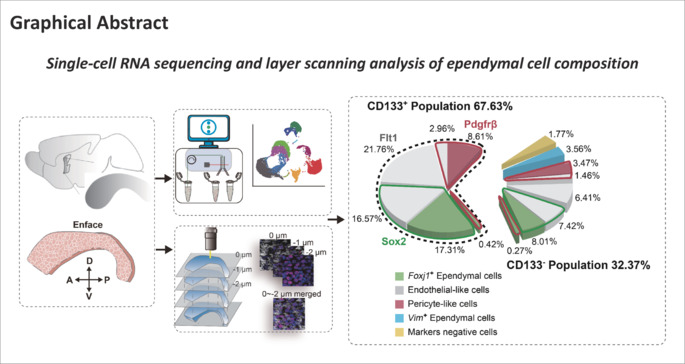

The presence or absence of adult neural stem cells in the mammalian forebrain ependyma has been debated for two decades. In this study, we performed single-cell RNA sequencing to investigate the cellular composition of the ependymal surface of the adult mouse forebrain using whole mounts of lateral walls of lateral ventricles. We identified 12 different cell subtypes in the ependymal surface. Immunocytochemical analyses revealed that CD133^+^ multi-ciliated cells comprised 67.6% of ependymal cells, while the remaining 32.4% were CD133^–^. CD133^+^ ependymal cells can be further classified into FOXJ1^+^/SOX2^+^/ACTA2^+^ cells, FLT1^+^/CD31^+^/CLDN5^+^ endothelial-like cells, and PDGFRB^+^/VTN^+^/NG2^+^ pericyte-like cells, as well as endothelial–pericyte-like cells and *Foxj1*^+^ endothelial-like cells. CD133^–^ ependymal cells can be further divided into endothelial-like cells, *Foxj1*^+^ ependymal cells, *Foxj1*^+^ endothelial-like cells, pericyte-like cells, endothelial-pericyte-like cells, VIM^+^ cells, and cells negative for all of these markers. This comprehensive profiling confirms the heterogeneity of the ependymal surface in the adult mouse forebrain. Debate regarding whether adult ependymal cells contain neural stem cells has arisen because different researchers have examined different populations of ependymal cells. Our study provides a new perspective for investigation of clinical endogenous neural stem cells, ultimately paving the way for stem cell therapies in neurological diseases.

## Introduction

Ongoing neurogenesis in the mammalian central nervous system (CNS) occurs in specific regions throughout life (Ming and Song, 2005). In rodents, the most well-characterized regions are the subgranular zone of the hippocampal dentate gyrus, which produces granule neurons, and the ependymal and subependymal zones lining the lateral ventricles, which continuously generate new neurons migrating along the rostral migratory stream to populate the olfactory bulb (Gage, 2000; Fuentealba et al., 2015; Obernier et al., 2018). However, in adult rodents, the presence of neural stem cells (NSCs) in ependymal cells has been controversial. *Notch1*^+^ ependymal cells in adult rats and mice were initially thought to have NSC activity (Johansson et al., 1999). Meanwhile, CD24^+^ ependymal cells have no NSC activity, as indicated by their inability to form self-propagating NSCs (Doetsch et al., 1999; Capela and Temple, 2002). Subsequent studies identifying actin α2 (*Acta2*) as an ependymal marker similarly found no NSC function in ependymal cells (Shah et al., 2018). Conversely, fluorescence-activated cell sorting (FACS)-purified multi-ciliated mouse CD133^+^ ependymal cells form propagating single cell-derived clonal neurospheres (Uchida et al., 2000); CD133 is a human embryonic stem cell marker. Despite this, CD24^+^ ependymal cells have consistently shown no NSC activity, regardless of CD133 positivity. In addition, certain subependymal glial fibrillary acidic protein (GFAP)^+^ B1 cells, which are reportedly very active NSCs, also present a single primary cilium and are CD133^+^ (Mirzadeh et al., 2008; Codega et al., 2014). They are, however, restricted to the forebrain and may still be derived from CD133^+^ ependymal cells.

Preliminary transcriptomic data from FAC-sorted prominin 1 (*Prom1*)^+^ (CD133) and *Prom1*^–^ cells have allowed the identification of additional characteristics of ependymal cells and B1 NSCs. Among *Prom1*^+^ cells, some were *Gfap* double positive (Codega et al., 2014), and these *Prom1*^+^/*Gfap*^+^ cells had lower *Prom1* expression than *Gfap*^–^ counterparts. This suggests that the transition from a *Prom1*^+^ to *Gfap*^+^ phenotype may be a potential B1 NSC feature. Transcriptomic features of *Prom1*^+^/*Gfap*^–^ cells were similar to endothelial cells, expressing vascular endothelial growth factor receptor 1 (*Vegfr* or *Flt1*), kinase insert domain receptor (*Kdr*), claudin-5 (*Cldn5*), and SRY-box transcription factor 17 (*Sox17*). A fraction of these cells also expressed platelet-derived growth factor receptor beta (*Pdgfrb*), a pericyte marker (Luo et al., 2015). These cells mitotically respond to vascular endothelial growth factor (VEGF), but not fibroblast growth factor 2 (*Fgf2*), yet can generate NESTIN^+^ and GFAP^+^ progenies that are FGF2 responsive (Luo et al., 2015). Although B1 NSCs are restricted to the forebrain, CD133^+^ ependymal cells are present throughout the entire ventricular surface, but are less mitotically active, as their primary mitogen is VEGF (Luo et al., 2015). If these ependymal cells are indeed NSCs, this would suggest that the entire mammalian CNS contains quiescent stem cells that can be activated by VEGF, an abundant factor during CNS injury.

Heterogeneity of the rodent forebrain is evident. The CD133^+^ subpopulation itself comprises both CD24^+^ and CD24^–^ subpopulations and potentially GFAP^+^ B1 NSCs (Doetsch et al., 1999; Capela and Temple, 2002; Codega et al., 2014). By extension, the neurogenic ependyma (i.e., the ependymal surface of lateral walls of lateral ventricles) must also be heterogeneous, containing diverse CD133^+^ and CD133^–^ populations. Previous conclusions regarding the absence of adult NSCs in the ependyma may have over-generalized their findings by the exclusion of other subpopulations (Doetsch et al., 1999; Johansson et al., 1999; Mirzadeh et al., 2008; Codega et al., 2014; Luo et al., 2015; Shah et al., 2018).

To set a robust foundation for future double or triple marker lineage tracing studies revisiting the question of whether the adult ependyma contains NSC activity, we examined the cellular composition and structures of en face preparations of the adult mouse neurogenic ependymal surface. We used single-cell RNA sequencing (scRNA-seq) and detailed immunocytochemical analyses.

## Methods

### Animals

All experimental procedures were approved by and performed according to the standards of the Animal Welfare Committees of Tongji University, Shanghai (approval No. TJAA06621106, March 4, 2021), China. Eight-week-old male and female C57BL/6J mice (Zhejiang Vital River Laboratory Animal Technology Co., Ltd., Tongxiang, Zhejiang, China; animal license No. SCXK (Zhe) 2020-0002) weighing between 18 and 20 g were used. Animals were housed in a specific pathogen-free breeding environment, with a controlled temperature of 22–25°C, humidity levels between 55%–60%, and a 12-hour light/dark cycle, typically with illumination ranging from 150–300 lx during the light phase. Mice were anesthetized by intraperitoneal injection with 0.8% pentobarbital (Sigma–Aldrich).

### Microdissection of lateral walls of lateral ventricles

Mice were sacrificed by cervical dislocation and the brains immediately removed. A thin layer of the lateral wall was submerged in fresh artificial cerebrospinal fluid (124 mM NaCl [Sigma–Aldrich, St. Louis, MO, USA, S3014], 2.5 mM KCl [Sigma–Aldrich, P9333], 1.2 mM NaH_2_PO_4_ [Sigma–Aldrich, S3139], 24 mM NaHCO_3_ [Sigma–Aldrich, S5761], 5 mM N-2-hydroxyethylpiperazine-N-2-ethane sulfonic acid [Sigma–Aldrich, S3375], 13 mM glucose [Sigma–Aldrich, G8270], 2 mM MgSO_4_ [Sigma–Aldrich, 746452], and 2 mM CaCl_2_ [Sigma–Aldrich, C5670]), pre-bubbled with oxygen (95% oxygen and 5% carbon dioxide) on ice. Whole mounts of lateral walls of lateral ventricles were dissociated as described (Carlén et al., 2009; Mirzadeh et al., 2010). These were en face preparations.

### Single cell preparation

Lateral walls of lateral ventricles were digested with papain (Worthington, Lakewood, NJ, USA, LS003126, 1 mg/mL, 30 minutes at 37°C) and DNase I (Worthington, LS006333) in 10 mL Hank’s balanced salt solution (Gibco, Grand Island, NY, USA, 14175-095). The solution was mixed every 5 minutes. Digested tissue was triturated into single cells in Hank’s balanced salt solution containing 1% bovine serum albumin (Sigma–Aldrich, B2064) and 3 µM actinomycin D (Sigma–Aldrich, A1410) using fire-polished Pasteur pipettes (five times with a 600 µm pipette, five times with a 300 µm pipette, and then 5–10 times with a 150 µm pipette). The cell suspension was filtered through a 40 µm cell strainer (Falcon, Durham, NC, USA, 352340), and then layered on top of 20% Percoll solution (GE Healthcare, Chicago, IL, USA, 17-0891-01). The mixture was centrifuged at 400 × *g* for 9 minutes at **~**20–25°C to remove debris and myelin. After Percoll density gradient centrifugation, high-quality single cells were enriched at the bottom. Pellets were resuspended in resuspension buffer (1% bovine serum albumin in Hank’s balanced salt solution) without actinomycin D. Cells were then immediately transferred to a new low-binding 2 mL tube (Eppendorf, Hamburg, Germany, 0030120094), incubated in resuspension buffer on ice for 5 minutes to a recovery state.

### CD133^+^ ependymal cells enriched by fluorescence-activated cell sorting

Single cell suspensions were incubated with isotype IgG-PE (eBioscience, San Diego, CA, USA, 12-4301-82) and CD133-PE antibody (eBioscience, 12-1331-82) in resuspension buffer on ice for 30 minutes. Cell solutions were then diluted into a suspension of 1000 cells/mL with phosphate buffered saline (PBS). CD133^+^ and CD133^–^ cells were collected using a gentle flow cytometry sorter at a pressure of 13.8 kPa. Wolf’s microfluidic channels enriched high-quality cells (Wolf Cell Sorter, NanoCellect, San Diego, CA, USA) (Jagnandan and Morachis, 2022).

### Single cell library preparation and sequencing

Both ‘PE’ (CD133^+^) and ‘NC’ (CD133^–^) cells were subjected to scRNA-seq using the 10× Genomics platform (Pleasanton, CA, USA). Single-cell captures and reverse transcription were performed as described previously (Lee et al., 2020; Liu et al., 2021). Single cell suspensions were diluted to 800–1000 cells/µL. The Chromium Single Cell 3ʹ Library, Gel Bead & Multiplex was used according to the manufacturer’s instructions for the Chromium Controller Kit (10× Genomics). Single cells were encapsulated into emulsion droplets to build a library for scRNA-seq. Single cell gel beads were loaded onto a Chromium controller (10× Genomics) for sequencing using a 75-cycle run kit Illumina HiSeq X platform (San Diego, CA, USA) with 150 bp paired-end reads.

### Pre-processing of scRNA-seq data

Raw reads were processed into gene expression profiles by CellRanger software (v7.0.1; 10x Genomics) (Zheng et al., 2017). Raw output data files (barcodes. tsv, features. tsv, matrix. mtx) were quantified by alignment to the Mm9 mouse reference genome (Bethesda, MD, USA). Raw data were analyzed using the Seurat (v4.4.0) package (Seattle, WA, USA) (Hao et al., 2021). During creation of the Seurat object, cells expressing < 200 genes and any genes expressed in < 5 cells were excluded. After calculating feature counts and mitochondrial gene ratios for each cell, additional filtering was performed. Cells with < 180 or > 8000 features were filtered out, as well as those with > 10% mitochondrial gene content. The DoubletFinder package was used to predict and remove potential double cells. A total of 14,658 cells were obtained for subsequent bioinformatics analyses after processing.

### Data integration, dimensionality reduction, cell clustering, and annotation of scRNA-seq data

Raw output data of each sample were read using the Read10× function in the Seurat package. The Seurat object for single-cell data analysis was created using the CreateSeuratObject function. After low-quality cell removal, data were normalized using the NormalizeData function. The vst method was used to identify the top 3000 highly variable genes, and principal components analysis dimensionality reduction was performed based on these 3000 highly variable genes. According to ElbowPlot, the optimal number of principal components is 10. Overall, 11 clustered populations of 14,658 cells were obtained using the FindNeighbors and FindCluster functions in the Seurat package. These were displayed by uniform manifold approximation and projection (UMAP) plots. To annotate cell clusters, different genes in the clusters were identified using the FindAllMarkers function with the default nonparametric Wilcoxon rank-sum test. Cell groups were annotated according to differentially expressed genes and known cell markers from the literature.

### Immunocytochemistry

Mice were anesthetized by intraperitoneal injection with 0.8% pentobarbital (Sigma–Aldrich) and sacrificed by intracardial perfusion of normal saline and 4% paraformaldehyde. Brains were extracted from the skull and post-fixed in 4% paraformaldehyde overnight, then transferred into 30% sucrose to dehydrate. Brains were embedded in optimal cutting temperature compound and 30 µm coronal sections cut using a cryostat (Leica, Wetzlar, Germany, VT1000S). Whole mounts were prepared as previously described. Whole mounts of lateral walls of lateral ventricles were dissociated and fixed in 4% paraformaldehyde overnight before staining.

Brain sections and whole mount preparations (as described in “Microdissection of lateral walls of lateral ventricles”) were incubated in blocking buffer (PBS with 3% bovine serum albumin, 2% normal donkey serum, and 0.5% Triton-X 100) for 2 hours and then incubated in primary antibodies in blocking solution for 16 hours at 4°C. After washing with PBS, sections and whole mounts were incubated with secondary antibodies and 4′,6-diamidino-2-phenylindole dihydrochloride (DAPI; Sigma–Aldrich, D9542) for 2 hours at **~**20–25°C, and then mounted on slides with mounting medium (glycerol, Mowiol 4-88, 0.2 M Tris buffer in distilled water). The antibodies used are detailed in **Additional Table 1**.

**Additional Table 1:** Information on antibodies

Antibody	Host	Dilution	Catalog number	RRID	Supplier
Anti-CD133	Rat	1:500	13-1331-82	AB_466591	eBioscience, San Diego, CA, USA
Anti-SRY-box transcription factor 2 (SOX2)	Rabbit	1:500	Ab92494	AB_10585428	Abcam, Cambridge, UK
Anti-SOX2	Goat	1:500	SC-17320	AB_2286684	Santa Cruz, Santa Luz, CA, USA
Anti-glial fibrillary acidic protein	Chicken	1:1000	PA1-10004	AB_1074620	Thermo Fisher, Waltham, MA, USA
Anti-epidermal growth factor receptor	Mouse	1:200	SC-373746	AB_10920395	Santa Cruz
Anti-Vimentin	Mouse	1:200	SC-6260	AB_628437	Santa Cruz
Anti-platelet derived growth factor receptor beta	Rat	1:200	AF1042-SP	AB_2162633	eBioscience
Anti-vascular endothelial growth factor receptor 1	Rabbit	1:200	ab2350	AB_303000	Abcam
Anti-Fltl	Mouse	1:500	SAB1405819	AB_10742048	Sigma-Aldrich
Anti-β-Catenin	Guinea pig	1:500	281004	AB_2661781	Synaptic Systems, Göttingen, Germany
Anti-CD31	Mouse	1:100	ab11939	AB_2132349	Abcam
Anti-Notch1	Rabbit	1:200	4380	AB_10691684	Cell Signaling Technology, Danvers, MA, USA
Anti-Nestin	Mouse	1:200	MAB1259	AB_2251304	R&D System, Minneapolis, MN, USA
Anti-Forkhead box J1	Rabbit	1:200	HPA005714	AB_1078902	Sigma-Aldrich, St. Louis, MO, USA
Anti-S100 calcium binding protein B	Rabbit	1:200	ab52642	AB_882426	Abcam
Anti-actin alpha 2	Rabbit	1:200	ab5694	AB_2223021	Abcam
Anti-vacuolar protein sorting-associated protein 35	Goat	1:100	ab10099	AB_296841	Abcam
Anti-cellular communication network factor 1	Rabbit	1:500	ab230947	AB_3107055	Abcam
Anti-CDl 1b/c	Mouse	1:1000	ab1211	AB_442947	Abcam
Anti-ionized calcium–binding adapter molecule 1	Rabbit	1:500	019-19741	AB_839504	Wako, Osaka, Japan
Anti-acetylated-tubulin	Mouse	1:1000	T6793	AB_477585	Sigma-Aldrich
Daylight 488 Lectin	-	1:500	DL-1174	AB_2336404	Vector, Burlingame, CA, USA
Alexa Fluor® 488 AffiniPure™ F(ab')_2_ Fragment Donkey Anti-Goat IgG (H+L)	-	1:1000	705-546-147	AB_2340430	Jackson ImmunoResearch Labs, West Grove, PA, USA
Alexa Fluor^®^ 488 AffiniPure™ F(ab')_2_ Fragment Donkey Anti-Rat IgG (H+L)	-	1:1000	715-546-150	AB_2340849	Jackson ImmunoResearch Labs
Alexa Fluor^®^ 488 AffiniPure™ F(ab')_2_ Fragment Donkey Anti-Chicken IgY (IgG) (H+L)	-	1:1000	703-546-155	AB_2340376	Jackson ImmunoResearch Labs
Alexa Fluor® 488 AffiniPure™ F(ab')_2_ Fragment Donkey Anti-Rabbit IgG (H+L)	-	1:1000	711-546-152	AB_2340619	Jackson ImmunoResearch Labs
Alexa Fluor® 647 AffiniPure™ F(ab')_2_ Fragment Donkey Anti-Rat IgG (H+L)		1:1000	712-606-153	AB_2340696	Jackson ImmunoResearch Labs
Alexa Fluor® 647 AffiniPure™ F(ab')_2_ Fragment Donkey Anti-Goat IgG (H+L)	-	1:1000	705-606-147	AB 2340438	Jackson ImmunoResearch Labs
Alexa Fluor® 647 AffiniPure™ F(ab')_2_ Fragment Donkey	-	1:1000	715-606-150	AB_2340865	Jackson ImmunoResearch Labs
Anti-Mouse IgG (H+L) Cy™3 AffiniPure™	-	1:1000	705-166-147	AB_2340413	Jackson ImmunoResearch
F(abʹ)2 Fragment Donkey Anti-Goat IgG (H+L) Cy™3 AffiniPure™	-	1:1000	712-166-153	AB_2340669	Labs Jackson ImmunoResearch
F(ab')2 Fragment Donkey Anti-Rat IgG (H+L) Cy™3 AffiniPure™	-	1:1000	715-166-150	AB_2340816	Labs Jackson ImmunoResearch
F(ab')2 Fragment Donkey Anti-Mouse IgG (H+L)					Labs

### Z-stack imaging of ependymal cells

Cilia are only observed in the top three optical layers; therefore, images of these layers were collected for subsequent quantitative analysis. Two methods were used to calculate the percentage of marker-positive cells in total 4′,6-diamidino-2-phenylindole dihydrochloride-positive cells. 1) Added method: images of the three layers (A: 0 µm, B: –1 µm, and C: –2 µm) were counted separately, then added and averaged; and 2) Merged method: projections of 0 µm, –1 µm, and –2 µm images were merged (D) and then counted. Both methods calculate mean values to approximate the actual cellular composition: Average 1 = (A + B + C + D)/4, and Average 2 = (A + B + C)/3 + D/2.

### Electron microscopy

En face tissue was collected as previously described and transferred to ice-cold PBS (Gibco, Cat# 11965092). Tissue was washed at 80 r/min in three wash cycles of 2, 5, and 3 minutes, respectively. Tissue was fixed overnight at 4°C in 1% glutaraldehyde solution (Sigma–Aldrich, Cat# G6257) and then washed five times in PBS on a shaker the next day. Osmic acid (Sigma–Aldrich, Cat# 1.24505) was added to fix the tissue for 1 hour, followed by four additional PBS washes. Sequential dehydration was performed using 30%, 50%, 70%, 90%, and 100% ethanol solutions (Sigma–Aldrich, Cat# 1.00986) for 10 minutes each, with an additional three rounds of dehydration using 100% ethanol. The en face preparation was then transferred to a cell culture dish and dried overnight in a 50°C incubator. Once dried, they were mounted onto the scanning electron microscope stage using gold sputtering tape and coated with gold. After stabilizing the negative pressure, imaging was performed using an electron microscope (Hitachi S-3400N, Tokyo, Japan).

### Statistical analysis

Values were obtained using GraphPad Prism version 8.0.1 (244) for Windows (GraphPad Software, Boston, MA, USA, www.graphpad.com). Data are displayed as mean ± standard deviation (SD). Comparisons between two groups were analyzed by Student’s *t*-test, and comparisons among multiple groups were analyzed by one-way analysis of variance followed by Tukey’s *post hoc* test.

## Results

### Examination of the cellular composition of adult mouse neurogenic ependyma en face preparations by scRNA-seq

**Figure 1:**
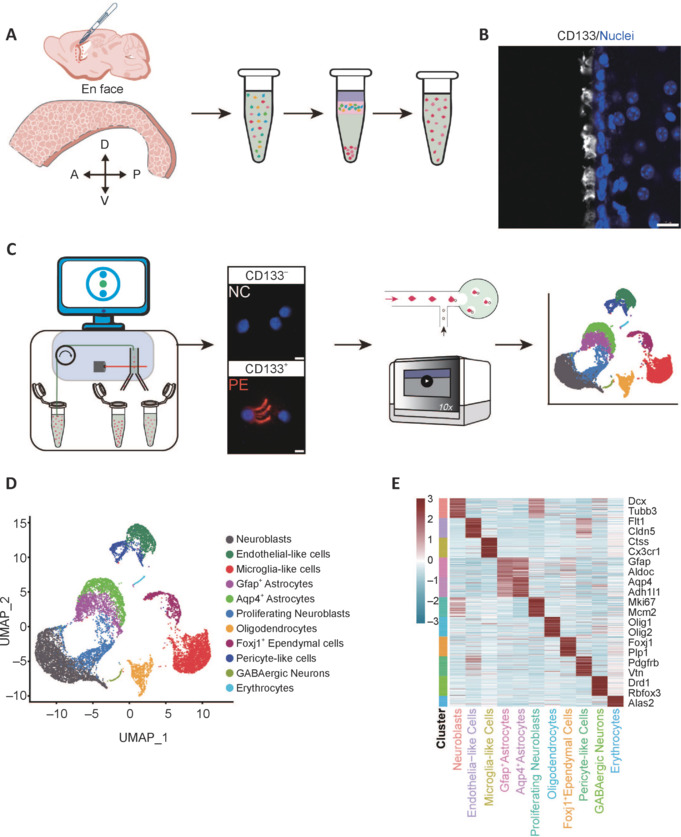
Single-cell sequencing showing the cellular composition of lateral walls of lateral ventricles (en face) in adult mice. (A) Whole mount tissue of lateral walls of lateral ventricles (en face) were dissected from the striatal wall (red outline). Single cells were dissociated from whole mount tissue and isolated by density gradient centrifugation. Image created using Adobe Illustrator CC 2018. (B) Immunocytochemical staining of CD133 (Alexa Fluor®647, white) in the lateral ventricle wall of adult mice. Scale bar: 15 μm. (C) CD133^+^ and CD133^–^ cells were enriched by gentle fluorescence-activated cell sorting. Scale bars: 5 µm. (D) Cells were annotated into 11 subtypes based upon single-cell RNA sequencing. (E) The top 50 highly expressed genes in each subpopulation and specific genes are shown in Additional Table 2. A: Anterior; D: dorsal; NC: CD133^–^ cells; P: posterior; PE: CD133^+^ cells; UMAP: uniform manifold approximation and projection; V: ventral.

Ependymal cells comprise the single layer of cells lining the ventricular surface, and as such are in direct contact with cerebrospinal fluid (Spassky et al., 2005; Del Bigio, 2010). Neurogenic en face preparations are whole tissue mounts of lateral walls of lateral ventricles, containing both ependymal and sub-ependymal structures and zones. To determine the cellular composition of adult mouse en face tissue preparations, with a focus on ependymal zones, we performed scRNA-seq (**Figure 1A**). En face tissue from 8-week-old C57BL/6J male mice (*n* = 7) was dissected and dissociated into single cells. As CD133 immunoreactivity (antibody Clone No. 13A4) specifically labels ependymal multi-ciliated cells, and particularly cilia (**Figure 1B** and **Additional Figure 1A**), ependymal cells with high CD133 protein expression were enriched by soft FACS. CD133^+^ cells were denoted as ‘PE’ cells and CD133^–^ cells were denoted as ‘NC’ cells (**Figure 1C**). High-quality scRNA-seq data were obtained from a total of 8594 cells in the NC fraction and 9017 cells in the PE fraction (**Additional Figure 1B**). Overall, 14,658 cells (7231 CD133^+^ cells and 7427 CD133^–^ cells) were obtained for subsequent bioinformatics analyses. In total, 11 cell clusters were annotated according to their specific gene signatures, including neuroblasts, proliferating neuroblasts, *Gfap*^+^ astrocytes, aquaporin 4 (*Aqp4*)^+^ astrocytes, oligodendrocytes, microglia-like cells, forkhead box protein J1 (*Foxj1*)^+^ ependymal cells, endothelial-like cells, pericyte-like cells, and erythrocytes (**Figure 1D**). The top 50 genes highly expressed in each cell subtype were relatively specific (Llorens-Bobadilla et al., 2015; Luo et al., 2015; Basak et al., 2018; **Figure 1E**, **Additional Figure 1C**, and **Additional Table 2**).

### The forebrain ependymal surface is mainly composed of endothelial-like cells, pericyte-like cells, and *Foxj1*^+^ ependymal cells

**Figure 2:**
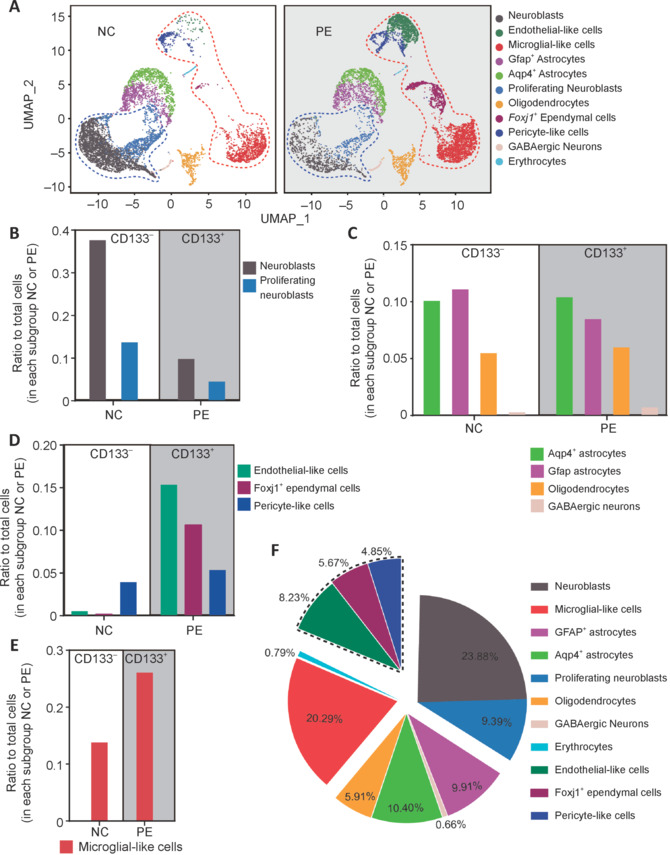
CD133 protein is enriched in endothelial-like cells, pericyte-like cells, and *Foxj1*^+^ ependymal cells. (A) Distribution of each subpopulation in CD133^+^ (PE) and CD133^–^ (NC) samples. Cells outlined in blue were enriched in the CD133^–^ sample, while cells outlined in red were enriched in the CD133^+^ sample. (B) The ratio to total cells (in each subgroup) of neuroblast and proliferating neuroblast clusters in CD133^+^ and CD133^–^ samples. (C) The ratio to total cells (in each subgroup) of *Aqp4*^+^ astrocytes, *Gfap*^+^ astrocytes, oligodendrocytes, and GABAergic neurons in CD133^+^ and CD133^–^ samples. (D) The ratio to total cells (in each subgroup) of endothelial-like cell, pericyte-like, and *Foxj1*^+^ ependymal cell clusters in CD133^+^ and CD133^–^ samples. (E) The ratio to total cells (in each subgroup) of microglia-like cells cluster in CD133^+^ and CD133^–^ samples. (F) The proportion of cells from different clusters among the total number of sequenced cells.

**Figure 3:**
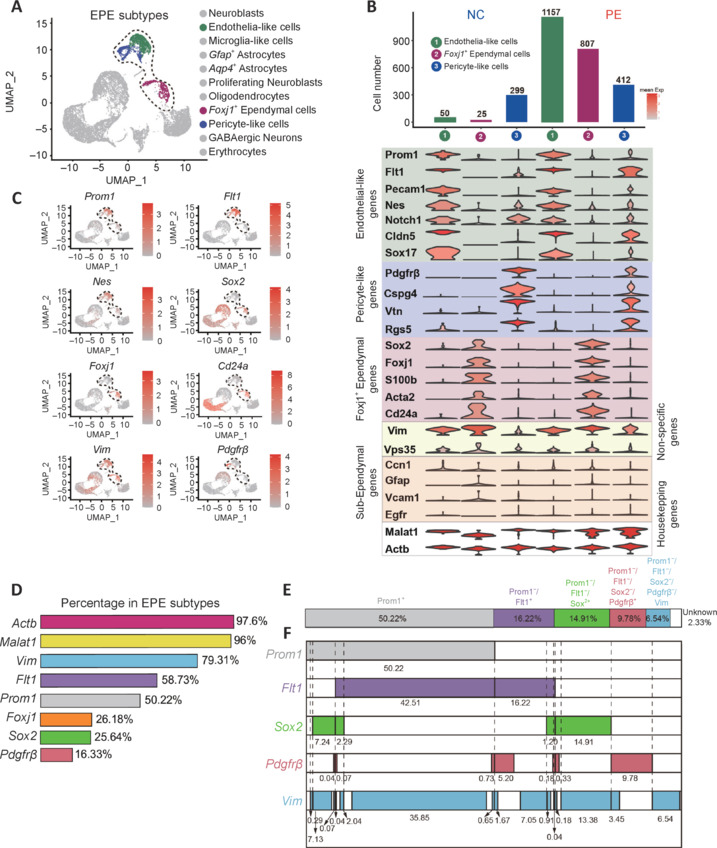
Composition of EPE cells by scRNA-seq. (A) The three subpopulations: endothelial-like cells, pericyte-like cells, and *Foxj1*^+^ ependymal cells were defined as EPE cells. (B) The upper section shows the number of cells in CD133^+^ (PE) and CD133^–^ (NC) samples of EPE cells. The lower section shows specific genes within these three clusters. Genes in the green box are mainly expressed in endothelial-like cells, in the blue box are highly expressed in pericyte-like cells, and in the red box are mainly expressed in *Foxj1*^+^ ependymal cells. Genes in the yellow box are expressed in all three EPE subtypes. (C) Expression profiles of the ependymal markers, *Prom1*, *Flt1*, *Nes*, *Sox2*, *Foxj1*, *Cd24a*, *Vim*, and *Pdgfrb*. EPE cells are outlined in black; and red spots indicate cells expressing target genes. (D) The proportion of ependyma in EPE cells expressing *Actb*, *Malat1*, *Vim*, *Flt1*, *Prom1*, *Foxj1*, *Sox2*, and *Pdgfrb* on. *Actb* and *Malat1* are housekeeping genes. (E) Cells in ependymal layers were categorized into five subgroups using a serial depletion method based on five representative EPE subtype-specific marker genes: *Prom1*, *Flt1*, *Sox2*, *Pdgfrb*, and *Vim*. (F) The distribution of cells co-expressing *Prom1*, *Sox2*, *Vim*, *Pdgfrb*, and *Flt1* in EPE cells. EPE cells: Endothelial-like cells, pericyte-like cells, and *Foxj1*^+^ ependymal cells.

CD133 was restricted to the ependyma, with its immunoreactivity specifically located on cilia (especially multi-cilia). The NC fraction, as expected, was enriched for neuroblasts and proliferating neuroblasts, indicating that anti-CD133 antibody does not detect these cells (**Figure 2A**). Previous studies have shown that these cells are present in the subependymal region and not the ependymal surface (Kelsch et al., 2010; Benner et al., 2013; **Figure 2A**, and **B**). Other groups of cells known to exist in the subependymal zone, including *Gfap*^+^ astrocytes, *Aqp4*^+^ astrocytes, and oligodendrocytes (Doetsch et al., 1999), were not enriched in either the NC or PE fractions. This suggests that CD133 expression levels in these cells was borderline positive/negative (**Figure 2A**, and **C**). *Gfap*^+^ astrocytes from the PE fraction are likely to be consistent with previously reported *Prom1*^+^/*Gfap*^+^ astrocytes. Endothelial-like cells, pericyte-like cells, and *Foxj1*^+^ ependymal cells (EPE cells) were enriched in the PE fraction (outlined with a red dotted line), indicating positive CD133 antibody labeling (**Figure 2A**, and **D**). Previous studies have shown that these groups of cells express high levels of CD133 protein. Microglia were also positively enriched in the PE fraction, accounting for 20.3% of total cells sequenced (**Figure 2A**, **E**, and **F**). Follow-up immunocytochemical staining did not detect microglia-like cells (IBA1^+^ and CD11B/C^+^) in meaningfully observable quantities within the ependyma (**Additional Figure 2A**). Therefore, it remains to be determined whether these microglia-like cells are in circulation or on the ependyma proper; and if the latter, where and what they are. Because these microglia-like cells are most likely not on the ependyma (also suggested by follow-up immunocytochemical analyses), they were excluded from further analyses. The number of EPE cells accounted for 18.8% of the total number of cells sequenced (**Figure 2F**). As the majority are highly positive for the cell surface marker, CD133, such groups of cells were designated as EPE cells for subsequent analyses (**Figures 2A**, **F**, and **3A**).

Based upon EPE cell numbers in both PE and NC fractions, EPE cells were found to be enriched in PE fractions (**Figure 2A** and **Additional Figure 2B**, **C**). PE samples (i.e., CD133^+^ ependymal cells) contained roughly 50% endothelial-like cells, 34% *Foxj1*^+^ ependymal cells, and 17% pericyte-like cells (**Figure 3B**). There were overlaps among these three major cell subtypes. Cells expressing both endothelial and pericyte markers were labeled as pericyte-like cells, and cells expressing both *Foxj1* and endothelial markers were labeled as *Foxj1*^+^ ependymal cells. Few, if any, cells co-expressed both *Foxj1* and *Pdgfrb*, suggesting separation of these two ependymal subtypes (**Figure 3B**, and **C**). We also observed that endothelial-like cells and *Foxj1*^+^ ependymal cells were highly enriched in the PE fraction, while pericyte-like cells showed less enrichment. This may suggest that pericyte-like cells present less cell surface CD133 immunoreactivity compared with the other two ependymal subtypes (endothelial-like and *Foxj1*^+^ ependymal cells) (**Figure 3B**).

Because there is heated debate on whether the mammalian ependyma contains adult NSCs, we investigated the expression levels of all previously published markers. Previous studies have used different markers to label adult NSCs, ependymal, and subependymal cells, reaching different conclusions. In EPE cells, these markers include: *Prom1*, *Flt1*, *Pecam1*, *Sox17*, *Cldn5*, *Nes*, *Notch1*, *Pdgfrb*, *Cspg4*, *Vtn*, *Sox2*, *Foxj1*, *S100b*, *Acta2*, *Cd24a*, *Vim*, *Vps35*, *Ccn1*, *Gfap*, *Vcam1*, and *Egfr*, together with two housekeeping genes, *Malat1* and *Actb* (**Figure 3B** and **Additional Figure 3**). As shown in **Figure 3B**, these markers co-segregated into six groups. The first group (endothelial-like) included *Prom1* (CD133), *Flt1*, *Pecam1* (CD31), *Sox17*, *Cldn5*, *Nes* (encodes Nestin), and *Notch1*. These were highly expressed in the endothelial-like subtype and pericyte-like cells, but not *Foxj1*^+^ ependymal cells. The second group (pericyte-like) included *Pdgfrb*, *Cspg4* (NG2), and *Vtn*, and were clearly pericyte-like cell-specific. The third group (*Foxj1*^+^ ependymal) included *Sox2*, *Foxj1*, *S100b*, *Acta2*, and *Cd24a*, and were specifically expressed in the *Foxj1*^+^ ependymal subtype. The fourth group comprised vacuolar protein sorting-associated protein 35 (*Vps35*) and vimentin (*Vim*). These markers were expressed in all three EPE subtypes, although pericyte-like cells showed less expression compared with the other two subtypes (**Figure 3B**). *Vps35* has previously been reported as a marker for ependymal cells, and its absence can lead to severe brain dysplasia and hydrocephalus (Wu et al., 2020b). Vim expression in the ventricular zone can enable plasticity and regenerative ability in the adult brain (Arochena et al., 2004; Morrow et al., 2020). The fifth group, comprising *Ccn1*, *Gfap*, *Vcam1*, and *Egfr*, is expressed in sub-ependymal adult NSCs (Hu et al., 2017; Wu et al., 2020a). The sixth group included housekeeping genes (**Figure 3B**). These results suggest that previous studies indicating that the ependyma does not contain adult NSCs have only studied one ependymal subtype, i.e., the *Foxj1*, *Cd24a*, and *Acta2* positive ependymal subtype, and not *Notch1*/*Flt1* positive nor *Cspg4*/*Pdgfrb* positive ependymal cells.

It is worth clarifying that co-segregation of the markers examined here refers only to EPE cells. Sequencing results also indicated, for example, that *Vim* was highly expressed in proliferating neuroblasts and *Gfap*^+^ astrocyte subgroups (**Figure 3C**). The expression patterns of these markers in the sub-ependymal region are shown in **Figure 3C** and **Additional Figure 4**, although the sub-ependymal zone was not the focus of this study.

### Reconstitution of the cellular subtype composition of ependymal cells based on scRNA-seq data

Based on scRNA-seq results, approximately 80% of EPE cells expressed *Vim* (**Figure 3D**). More than 50% of EPE cells expressed *Flt1* or *Prom1*, around 25% expressed *Sox2*, 25% expressed *Foxj1*, and around 16% expressed *Pdgfrb* (**Figure 3D** and **Additional Figure 5**). Because of significant levels of co-expression, a serial depletion method was used based on five representative EPE subtype-specific marker genes: *Prom1*, *Flt1*, *Sox2*, *Pdgfrb*, and *Vim*. EPE cells could then be grouped into five subpopulations: 50.2% *Prom1*^+^ cells, 16.2% *Prom1*^–^/*Flt1*^+^ cells, 14.9% of *Prom1*^–^/*Flt1*^–^/*Sox2*^+^ cells, 9.7% *Prom1*^–^/*Flt1*^–^/*Sox2*^–^/*Pdgfrb*^+^ cells, and 6.5% of *Prom1*^–^/*Flt1*^–^/*Sox2*^–^/*Pdgfrb*^–^/*Vim*^+^ cells (**Figure 3E**, and **F**). The above five subpopulations of cells constituted 97.7% of EPE cells, and the remaining 2.3% of undefined cells only expressed the housekeeping genes, metastasis associated lung adenocarcinoma transcript 1 (*Malat1*) and actin (*Actb*) (**Figure 3E**, **F** and **Additional Figures 5A–C** and **6A–C**).

According to gene co-expression, the major gene of endothelial-like cells was *Flt1*, with 72.3% of *Flt1*^+^ cells also *Prom1*^+^ (**Figure 3F** and **Additional Figure 5D**). Approximately 37% of *Sox2* expressing *Foxj1*^+^ ependymal cells also expressed *Prom1*, whereas only 5% of pericyte-like cells expressed *Prom1* (**Figure 3F** and **Additional Figure 5D**). *Prom1*^+^ cells can be divided into two major groups: *Prom1*^+^/*Sox2*^+^ (19%) and *Prom1*^+^/*Flt1*^+^ (85%), with about 4%–5% overlap (i.e., *Prom1*^+^/*Flt1*^+^/*Sox2*^+^). Pericyte-like cells expressed *Pdgfrb*, *Cspg4*, and *Vtn*. *Pdgfrb*^+^ cells rarely co-expressed *Sox2* or *Vim* (**Figure 3F** and **Additional Figure 5D**). Approximately 38% of *Pdgfrb*^+^ cells co-labeled with *Flt1*, suggesting that there was a group of endothelial–pericyte (endo–peri)-like cells (**Figure 3F** and **Additional Figure 5D**). Approximately 13% of endo–peri-like cells are *Prom1*^+^. *Foxj1*^+^ ependymal cells mainly expressed *Foxj1* and *Sox2*. Around 24% of *Sox2*^+^/*Prom1*^+^ cells also express *Flt1* (**Figure 3F** and **Additional Figure 5A** and **B**). *Sox2* and *Pdgfrb* were rarely expressed simultaneously in any cells, but *Vim*^+^ cells were often co-labeled with *Prom1*, *Sox2*, and *Flt1*. This indicates that *Sox2*, *Flt1*, and particularly, *Pdgfrb*, are relatively EPE cell-specific subtype markers, while *Vim* is not (**Figure 3F** and **Additional Figure 5D**). Along with *Prom1*, *Flt1*, *Sox2*, and *Pdgfrb* markers being negative, the *Vim*^+^ population comprised about 6.5% of EPE cells. From this scRNA-seq analysis, neurogenic en face ependymal cells were categorized into *Prom1*^+^ and *Prom1*^–^ categories, and in each category, there were three major subtypes: endothelial-like (*Flt1*/*Pecam1*/*Sox17*/*Nes*/*Notch1* positive), *Foxj1*^+^-ependymal (*Foxj1*/*Sox2*/*Acta2*/*S100b*/*Cd24a* positive), and pericyte-like (*Pdgfrb*/*Cspg4*/*Vtn* positive). There was also two minor subtypes: endo–peri-like (*Flt1* and *Pdgfrb* double positive) and *Foxj1*–endo-like (*Flt1* and *Foxj1* double positive). In the *Prom1*-category, there was also a small *Vim*^+^ population. These 11 subtypes comprised 97.7% of EPE cells (**Figure 3E** and **Additional Figures 5A** and **6A–C**).

### Neurogenic en face ependymal cell subtype composition validated and refined by immunocytochemical staining

**Figure 4:**
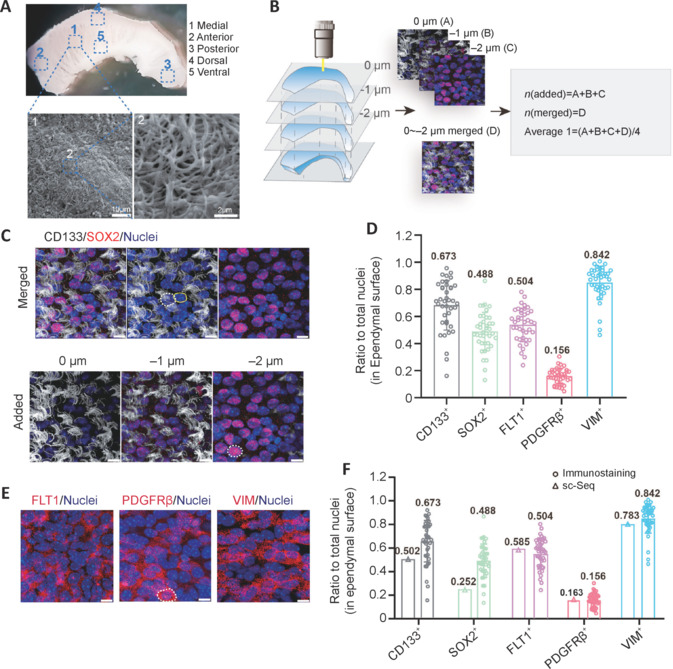
Whole-mount immunocytochemical staining of en face preparations showing the cellular composition of ependyma. (A) Cilia of ependymal cells were observed from a ventricular view using a scanning electron microscope. (B) The composition of ependymal cells was observed by confocal scanning of layer-by-layer Z-stacking (interval, 1 µm). From a ventral aspect, the Z-stack depths were: first layer was 0 µm, second layer was –1 µm, and third layer was –2 µm. Two methods were used to analyze positive rates: Method 1 (or Added): images from these three layers were counted individually and then mean values calculated; and Method 2 (or Merged): positive rates were counted from projections of these three layers. Average 1: All positive rates were used to calculate mean values. (C) Confocal microscopy of whole mounts. Added images from single confocal scans of the three layers (0 µm layer 1, –1 µm layer 2, and –2 µm layer 3). Whole mounts were stained by CD133 (Alexa Fluor®647, white), SOX2 (Cy^TM^3, red) and nuclei (4′,6-diamidino-2-phenylindole dihydrochloride, blue). Merged images were projections of the three layers viewed from the ventricular surface. Scale bars: 10 µm. (D) The ratio to total nuclei (in ependymal surface) of ependymal cells expressing CD133, SOX2, VIM, PDGFRβ, and FLT1 by whole-mount immunocytochemical staining. (E) Projection images of the three layers by whole-mount staining of FLT1 (Cy^TM^3, red), PDGFRβ (Cy^TM^3, red), and VIM (Cy^TM^3, red). White dotted circles represent cells expressing FLT1, PDGFRβ, or VIM; and yellow solid circles represent cells not expressing FLT1, PDGFRβ or VIM. Scale bars: 10 µm. (F) The ratio to total nuclei (in ependymal surface) of ependymal cells expressing CD133, SOX2, FLT1, PDGFRβ, and VIM on quantified using whole-mount immunocytochemical staining and single-cell RNA sequencing (sc-Seq).

Because scRNA-seq data contain no spatial information, analysis of EPE cells was based on prior knowledge of the ependyma. To validate our scRNA-seq-based ependymal cell subtype composition analysis, we next performed immunocytochemical staining using en face preparations. We examined the five markers, *Prom1* (CD133), *Sox2*, *Flt1*, *Pdgfrb*, and *Vim*, with their corresponding widely used antibodies (Coskun et al., 2008; Morrow et al., 2020; Delgado et al., 2021; Zhao et al., 2022). Electron microscopy showed that the lateral wall appeared rugged, rather than a flat and smooth structure (**Figure 4A**, ventricular view). Cilia of ependymal cells were distributed in clusters across the entire surface of the lateral wall (en face ependyma). Immunocytochemical staining detailing the cilia and outermost ependymal cells were captured in z-stacked images by laser confocal microscope (1 µm intervals) (**Figure 4B**).

Using the “Added method,” most CD133^+^ cilia were detected in 0 and –1 µm layers, while most SOX2 proteins appeared in –1 and –2 µm layers. Nuclei exhibited clear morphology in all three layers (**Figure 4C**). Protein expression at different depths from the surface were detected more accurately using the “Added image method,” while protein expression of the ependyma was observed more holistically using the “Merged image method” (**Figure 4C**). Immunocytochemical staining with five well-characterized primary antibodies (against CD133, SOX2, FLT1, PDGFRβ, and VIM) was used to systematically investigate ependymal cell subtype compositions (**Figure 4D** and **E**). To achieve more accurate marker positive rates, each marker positive rate was first calculated using both the Added and Merged methods (**Additional Figure 7**). Both Average 1 and Average 2 methods were used to obtain averaged rates of marker-positive subtypes of cells (**Additional Figure 7A**), which were found to be similar. Because values from the Average 1 method correlated better with the positive rates determined by single-cell transcriptome sequencing, this method was selected for the remaining analyses (**Figure 4D** and **Additional Figure 7**). Accordingly, 67% of all en face ependymal cells were CD133^+^, 49% were SOX2^+^, 50% were FLT1^+^, 16% were PDGFRβ^+^, and 84% were VIM^+^ (**Figure 4D**).

Immunocytochemical staining results were further compared with sc-RNA sequencing results. Both methods were highly correlated, but not identical (**Figure 4F**). For example, immunocytochemical staining of CD133, SOX2, and VIM protein marker positive rates were higher compared with those obtained from scRNA-seq data (**Figure 4F**), yet the opposite was found for FLT1. It is worth noting that immunocytochemical staining represents measurements based on protein levels, while scRNA-seq represents mRNA levels. Therefore, these two parameters do not necessarily have to be similar. Moreover, the lower mRNA capture rate associated with the 10× Chromium scRNA-seq platform may lead to false negatives. Conversely, immunocytochemical staining is affected by antibody quality and detection thresholds. Because immunocytochemical staining contains spatial information, as well as protein expression levels, it is considered a more accurate method for ependymal cell subtype composition analyses. Moreover, scRNA-seq provided superior, unbiased overall guidance on comprehensive immunocytochemical staining analyses.

**Figure 5:**
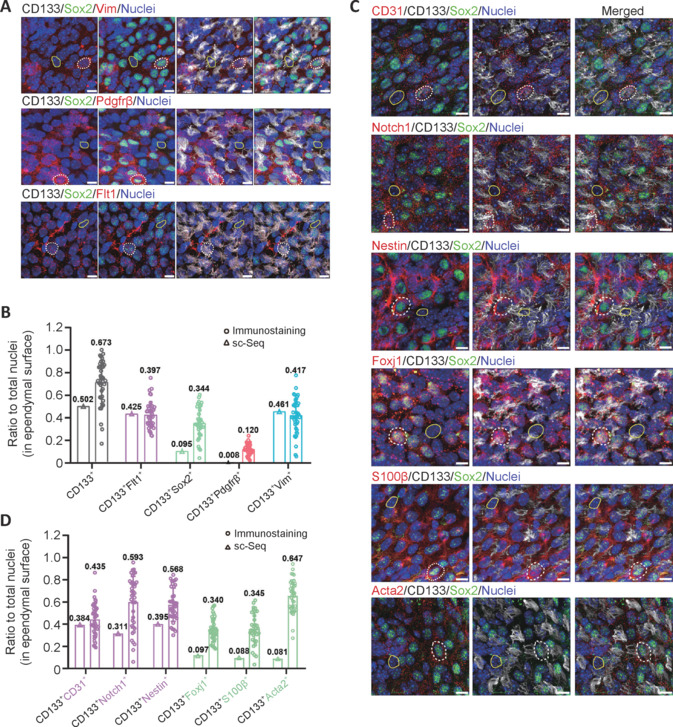
Co-expression ratio of ependymal cell markers by immunocytochemical staining. (A) Projection images of the three layers by whole-mount staining: CD133 (Alexa Fluor®647, white), SOX2 (Alexa Fluor®488, green), VIM (Cy^TM^3, red), PDGFRβ (Cy^TM^3, red), and FLT1 (Cy^TM^3, red). White dotted circles represent cells expressing VIM, PDGFRβ, or FLT1, and yellow solid circles represent cells not expressing VIM, PDGFRβ, or FLT1. Scale bars: 10 µm. (B) The ratio to nuclei (in ependymal surface) of CD133^+^, CD133^+^/SOX2^+^, CD133^+^/VIM^+^, CD133^+^/PDGFRβ^+^, and CD133^+^/FLT1^+^ cells quantified from whole-mount immunocytochemical staining and scRNA-seq. (C) Immunocytochemical staining of CD31, NOTCH1, NESTIN, FOXJ1, S100β, ACTA2 (Cy^TM^3, red), and CD133 (Alexa Fluor®647, white) and SOX2 (Alexa Fluor®488, green). White dotted circles represent cells expressing CD31, NOTCH1, NESTIN, FOXJ1, S100β, and ACTA2. Yellow solid circles represent cells not expressing CD31, NOTCH1, NESTIN, FOXJ1, S100β, or ACTA2. Scale bars: 10 µm. (D) Co-labeling ratios of CD133 and ependymal cell markers quantified by whole-mount immunocytochemical staining and single-cell RNA sequencing (scRNA-Sep).

Immunocytochemical staining with single markers suggested the presence of co-expression, as the percentages summed up to > 100%. Co-expression of these five markers was also apparent from scRNA-seq data. Thus, double and triple labeling was performed in immunocytochemical staining analyses. CD133 was still used as the first partition marker, and cells were co-stained for CD133 and SOX2, or FLT1, or PDGFRβ, or VIM (**Figure 5A**, **B** and **Additional Figure 8A**). The population of CD133 and FLT1 double-positive cells comprised 40% of all cells on the en face ependymal surface. The CD133^+^/SOX2^+^ population occupied 34%, CD133^+^/PDGFRβ^+^ population 12%, and CD133^+^/VIM^+^ population 42% (**Figure 5B**). Compared with the scRNA-seq data, the rankings on abundance of double positive cell populations were largely the same, while immuno co-labeling for SOX2/CD133 double positive and PDGFRβ/CD133 double positive populations tended to produce higher cell content than sc-RNA seq results. As already mentioned, scRNA-seq results might have false negatives, which could explain this discrepancy.

**Figure 6:**
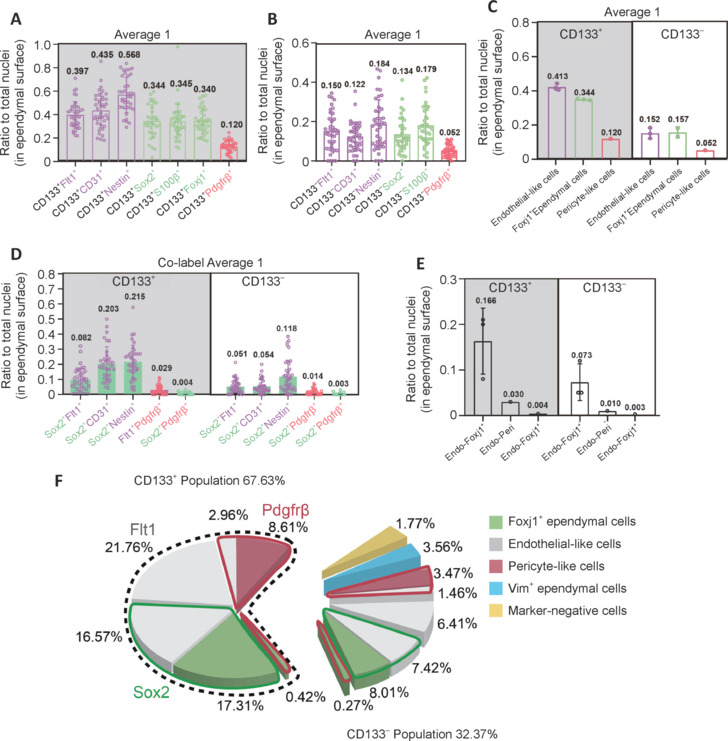
Cell subtype composition of the entire en face preparation. (A) The ratio to total nuclei (in ependymal surface) of FLT1, CD31, NESTIN, SOX2, S100β, FOXJ1, and PDGFRβ in CD133^+^ samples calculated by the Average 1 method. (B) The ratio to total nuclei (in ependymal surface) of FLT1, CD31, NESTIN, SOX2, S100β, and PDGFRβ in CD133^–^ samples calculated by the Average 1 method. (C) The ratio to total nuclei (in ependymal surface) of endothelial-like cells, pericyte-like cells, and *Foxj1*^+^ ependymal cells in CD133^+^ and CD133^–^ samples calculated by the Average 1 method. (D) The ratio to total nuclei (in ependymal surface) of FLT1, CD31, NESTIN, SOX2, and PDGFRβ co-labeling in CD133^+^ and CD133^–^ samples calculated by the Average 1 method. (E) The ratio to total nuclei (in ependymal surface) of endothelial-like, pericyte-like, and *Foxj1*^+^ ependymal subgroup co-labeling in CD133^+^ and CD133^–^ samples calculated by the Average 1 method. (F) Pie chart showing cell subtype composition of the entire ependyma. Cell proportions were calculated using the Average 1 method in CD133^+^ and CD133^–^ samples. Cells in the green sector express *Foxj1*^+^ ependymal cell-related genes and in the red sector express pericyte-like cell-related genes. The white sector represents endothelial-like cells; green sector represents *Foxj1*^+^ ependymal cells; red sector represents pericyte-like cells; blue sector represents VIM^+^ ependymal cells; and yellow sector represents ependymal cells express housekeeping genes.

In addition to these five representative markers, which labeled endothelial-like (FLT1^+^), *Foxj1*^+^, and pericyte-like CD133^+^ and CD133^–^ ependymal populations, other markers co-expressed in endothelial-like ependymal cells were investigated (CD31, NESTIN, and NOTCH1), as well as those co-expressed in *Foxj1*^+^-like cells (FOXJ1, S100β, and ACTA2) (**Figure 5C**, **D** and **Additional Figure 8B**). The overall data for endothelial markers were similar to FLT1, and for *Foxj1*^+^-like cells with respect to SOX2 and S100β. For ACTA2, levels were higher compared with SOX2 and FOXJ1. Although the antibodies were selected from previously published research, immunolabeling is highly dependent on the antibody quality. To ground the data collection, three markers (FLT1, CD31, and NESTIN) were used to label the endothelial-like-subgroup, and three markers (SOX2, FOXJ1, and S100β) for the *Foxj1*^+^-like subgroup (**Figure 6A**, **B** and **Additional Figure 8C**). Labeling results were averaged from these two sets of three markers to obtain subtype cell compositions of en face ependyma, CD133^+^ endothelial-like, and *Foxj1*^+^-like populations. Pericyte-like ependymal cell percentages were also calculated, as well as those for CD133^–^ endothelial-like, *Foxj1*^+^-like, and pericyte-like ependymal cells (**Figure 6C**). Because the total percentages of these six subtypes still exceeded 100%, there were again likely overlaps among them. To calculate the fractions of cells expressed in endothelial-like subgroup, *Foxj1*^+^-like subgroup, and pericyte-like subgroup markers, further co-labeling experiments were performed (**Figure 6D**, **E** and **Additional Figure 8D**). Fractions were counted for *Foxj1*^+^-endothelial-like, endothelial–pericyte-like, and *Foxj1*^+^-pericyte-like cells in both CD133^+^ fractions and CD133^–^ fractions. Finally, a pie chart illustrated the comprehensive cell subtype composition of the entire en face neurogenic ependyma, with 67.6% being CD133^+^ multi-ciliated cells. Within this population, there were five main subgroups: endothelial-like (FLT1^+^), *Foxj1*^+^ endothelial-like (FLT1^+^/SOX2^+^), *Foxj1*^+^ (SOX2^+^), pericyte-like (PDGFRβ^+^), and endo–peri-like (FLT1^+^/PDGFRβ^+^). Additionally, 32.4% were CD133^–^, comprising six major subgroups: endothelial-like, *Foxj1*^+^, *Foxj1*^+^ endothelial-like, pericyte-like, endo–peri-like cells, VIM^+^ only cells, and cells with all known markers negative (**Figure 6F** and **Additional Figure 9A**, **B**). Collectively, these cell subpopulations formed the whole (100%) of the neurogenic en face ependyma.

### Gene and protein distribution within the ependyma

Spatial positioning of the expressed proteins in the ependyma was also determined. CD133 and acetylated tubulin protein were predominantly localized in the cilia layer (**Additional Figure 8E**), where cilia are found in direct contact with cerebrospinal fluid. Gamma-tubulin (**Additional Figure 8F**), FOXJ1, ACTA2, S100Β, CD31, and NESTIN were localized in the apical layer, the layer between the cilia and nucleus. In the nuclear layer, the predominant proteins were VIM, CCN1, FLT1, CD31, and PDGFRβ. In the basal layer (bottom layer of ependymal cells, where they are in close contact with subependymal cells), VIM/CCN1/FLT1/CD31/PDGFRβ were highly enriched. In the subependymal layer, highly enriched with proliferating cells and neuroblasts, the processes of some GFAP^+^ cells extended cilia into the ependyma, with one or two CD133^+^ cilia in cases (**Additional Figure 9C**).

## Discussion

Arguments asserting the absence of NSCs in the adult rodent forebrain ependyma have over-generalized the findings because of a lack of perspective. This study is the first comprehensive profiling of mouse adult neurogenic ependymal cell subtype compositions, guided by detailed scRNA sequencing and validated and refined by extensive immunocytochemical analyses. All en face forebrain ependymal surface cell populations were characterized and quantified.

Previous studies claiming that the adult mouse ependyma does not contain adult NSCs, even when using multiple different markers (such as *Cd24a*, *Foxj1*, and *Acta2*), likely only studied the *Foxj1*^+^ or *Foxj1*^+^ endothelial-like CD133^+^ or CD133^–^ subpopulations of ependymal cells (Calaora et al., 1996; Meletis et al., 2008; Shah et al., 2018). As shown in this study, this does not represent the entire ependymal population. In contrast, previous studies claiming that ependyma contains adult NSCs have used *Notch1*, *Prom1* (CD133), and *Flt1* to label ependymal cells (Johansson et al., 1999; Corti et al., 2007; Coskun et al., 2008; Luo et al., 2015; Frederico et al., 2022). Based upon our findings, it is likely that both sides of this debate have studied different ependymal cell populations. Previous studies have shown that *Cd24a*^+^ ependymal cells, regardless of whether or not they are CD133^+^, do not manifest adult NSC activities, suggesting that such activities are absent in *Foxj1*^+^ endothelial-like cells (Coskun et al., 2008). Endothelial-like and endo–peri-like cells are likely to have adult NSC activities, but require VEGF, not basic FGF, as a mitogen (Luo et al., 2015). These cells are not restricted to the forebrain ependyma, but exist throughout the whole ventricular surface, including the third and fourth ventricles and central canal of the spinal cord (Martens et al., 2002). Whether pericyte-like ependymal cells contain adult NSC activities is still unclear. There are a number of lineage tracing studies using *Pdgfrb* promoter to drive Cre, showing neural lineage development in adult mice (Mo et al., 2020; Delgado et al., 2021). The role of *Gfap*^+^/*Pdgfrb*^+^ cells as adult NSCs has been proposed (Delgado et al., 2021). However, if *Pdfgrb* and *Prom1* double lineage tracing demonstrate neural lineage (neurons and glia) development in adult mice outside of the lateral ventricles (such as in the third and fourth ventricles or central canal), then pericyte-like and/or endo–peri-like cells may contain adult NSC activities as well. Because B1/2 cells do not exist in regions other than the forebrain ependyma, subependymal B1/2 cell contamination would not be an issue.

Based on previously published scRNA-seq data, the transcriptomes of ependymal cells in adult mouse spinal cord tissue before and after injury were reanalyzed (Li et al., 2022) (**Additional Figure 10**). Upon reexamination, the expression profiles of these spinal ependymal cells were notably different from their forebrain counterparts. *Prom1*^+^ endothelial-like and pericyte-like cells, likely surrounding the central canal, expressed higher levels of *Pecam1*, *Nes*, *Notch1*, and *Sox17*, suggesting that these pericyte-like cells are possibly endo–peri-like cells. Another notable difference was the expression of *Acta2*, which is specific to forebrain *Foxj1*^+^ ependymal cells, but not their spinal cord counterparts (**Additional Figure 11A** and **B**). Given that spinal cord *Foxj1*^+^ ependymal cells are postulated to present NSC activities after injury, whereas forebrain ependymal *Foxj1*^+^ cells lack NSC features, the potential role of *Acta2* in eliminating stemness from *Foxj1*^+^ forebrain en face ependymal cells warrants further investigation. Moreover, this also indicates that there are regional specificities within the entire CNS ependymal surface (Mizrak et al., 2019; Cebrian-Silla et al., 2021).

During development, *Sox2* and *Nes* are two well-known neural stem/progenitor cell markers (Graham et al., 2003; Ferri et al., 2004; Tobin et al., 2019). However, these two markers segregate in the adult neurogenic forebrain en face ependyma. *Sox2* is found in *Foxj1*^+^ ependymal cells, whereas Nes is expressed in *Flt1*^+^ endothelial-like ependymal cells. The former lacks NSC activity while the latter have shown NSC activities. There are a number of studies demonstrating that adult *Foxj1*^+^ ependymal cells are derived from embryonic ventricular zone neural stem/progenitor cells (*Glast*^+^ radial glia) (Gregg and Weiss, 2003; Spassky et al., 2005; Mirzadeh et al., 2017; Redmond et al., 2019). How these cells lose stemness remains to be determined. Moreover, how endothelial-like and peri-like ependymal cells differentiate and proliferate within the ependyma during postnatal development is also an important question that requires further investigation.

The discrepancy between scRNA-seq and immunocytochemical analyses is worthy of further consideration. As mentioned above, false negatives are an important concern for scRNA-seq technology because of relatively low mRNA capture rates (< 30%) (Zheng et al., 2017). However, immunocytochemical analyses can only be done with known markers and are limited by antibody quality (sensitivity and specificity) and counting methods. The two methods are complementary. Thus far, data obtained from both methods reinforce each other in aspects of overall cell subtype composition and abundance ranking. For endo–peri-like cells, immunocytochemical staining only demonstrates marker co-expression, and whether such cells are more endothelial-like or pericyte-like, can only be judged by transcriptomic data. From UMAP plots, some endo–peri-like cells are in the endothelial cluster and some are in the pericyte cluster, indicating potential subdivisions. Nevertheless, the endothelial and pericyte clusters are in close proximity, suggesting that these two types of cells are closely related based on gene expression patterns. The same situation is also true for *Foxj1*^+^ endothelial-like cells, in that double-positive cells are distributed in both *Foxj1*^+^ and endothelial clusters. In this case, the *Foxj1*^+^ cluster is quite different from the endothelial cluster (**Additional Figure 11C**). It is also worth noting that the corresponding three major subtypes of cells in the *Prom1*^+^ and *Prom1*^–^ populations (i.e., endothelial-like, pericyte-like, and *Foxj1*^+^), although belonging to the same clusters on UMAP plots, can still be separated or subdivided (**Additional Figure 12**). This suggests that they are meaningfully different in gene expression profiles. The resolution from scRNA-seq data alone is insufficient to comprehensively reveal core biological differences between CD133^+^ and CD133^–^ subclasses of ependymal cells.

Our data confirm that the adult mouse neurogenic ependyma is highly heterogeneous, containing at least 12 distinct subtypes overall that were divided by the 67.6% CD133^+^ (*Prom1*) populations and 32.4% CD133^–^ populations. Both CD133^+^ and CD133^–^ ependymal cells can be further divided into five and seven subpopulations, respectively. Within the CD133^+^ ependymal populations, which have adult NSC activities, there were at least five subpopulations: *Flt1*^+^/*Pecam1*^+^/*Sox17*^+^/*Notch1*^+^/*Nes*^+^ endothelial-like ependymal cells, *Foxj1*^+^/*Sox2*^+^/*Cd24a*^+^/*S100b*^+^/*Acta2*^+^ ependymal cells, *Pdgfrb*^+^/*Vtn*^+^/*Cspg4*^+^ pericyte-like ependymal cells, and two overlapping populations, endo–peri-like and *Foxj1*^+^ endothelial-like ependymal cells. The CD133^–^ ependymal population included two more subtypes: *Vim*^+^ only and negative for all known markers.

This study further illustrates the heterogeneity of cell subtypes in the adult mouse neurogenic forebrain ependyma. Based on these results, endothelial-like, pericyte-like, and endo–peri-like cells likely do present adult NSC activities. Conversely, *Foxj1*^+^ ependymal cells in this region likely do not harbor adult NSC features. Future lineage tracing studies with double or triple marker labeling alongside *in vivo* proliferation recording techniques such as ProTracer will be needed to more conclusively determine whether and which ependymal subtypes of cells contain NSC characteristics. The data and cell type analyses from this study will serve as a foundation for further investigations into whether the ependyma contains NSC activities. This study also offers new perspectives on the activation of endogenous neural stem cells in clinical patients, paving the way for stem cell therapies.

## Additional files:

***Additional Figure 1:*** *Single-cell RNA sequencing showing the cellular composition of en face preparations in adult mice.*

Additional Figure 1 Single-cell RNA sequencing showing the cellular composition of en face preparations in adult mice.
(A) CD133 (Alexa Fluor®647, white) was specifically expressed in lateral and apical walls of the lateral ventricle. Scale bar: 200 μm. (B) Quality control for single-cell transcriptomes of CD133+ (PE) and CD133- (NC) samples. (C) Specific marker genes were expressed by each subpopulation. The shade of red and diameter of spots represent gene expression levels.

***Additional Figure 2:*** *Single-cell RNA sequencing showing specific gene characteristics in EPE cells.*

Additional Figure 2 Single-cell RNA sequencing showing specific gene characteristics in EPE cells.
(A) CD11b/c+ (Cyanine Cy™3, red) and IBA1+ (Alexa Fluor®488, green) microglia were not detected in the lateral wall of the lateral ventricle in meaningfully observable quantities. Scale bars: 200 μm (left), 10 μm (right). (B) EPE cells were enriched in PE fractions. Red dots represent EPE cells in PE samples, and blue dots represent EPE cells in NC samples. (C) Expression of specific marker genes in EPE cells. Red spots represent gene expression. EPE cells: endothelial-like cells, pericyte-like cells, and Foxj1+ ependymal cells; NC: CD133- cells; PE: CD133+ cells.

***Additional Figure 3:*** *Single-cell RNA sequencing showing specific gene characteristics in NC and PE samples.*

Additional Figure 3 Single-cell RNA sequencing showing specific gene characteristics in NC and PE samples.
Expression of *Foxj1*^+^ ependymal cell-specific genes (*Sox2, Foxj1, S100b, Acta2*, and *Cd24a*), endothelial-like cell-specific genes (*Prom1, Flt1, Pecam1, Nes, Notch1, Cldn5*, and *Sox17*), pericyte-like cell-specific genes (*Pdgfrb, Cspg4, Vtn*, and *Rgs5*), other ependymal-related genes (*Vim, Vps35, Ccn1, Gfap, Egfr*, and *Vcam1*), and housekeeping genes (*Actb* and *Malat1*) in NC and PE samples. Red spots represent gene expression and EPE cells are outlined in black. EPE cells: Endothelial-like cells, pericyte-like cells, and *Foxj1*^+^ ependymal cells; NC: CD133- cells; PE: CD133^+^ cells.

***Additional Figure 4:*** *Characteristics of specific genes in each subgroup.*

Additional Figure 4 Characteristics of specific genes in each subgroup.
Expression of ependymal cell-specific genes in each cell subpopulation. Red spots represent expression of specific genes and EPE cells are outlined in black. EPE cells: Endothelial-like cells, pericyte-like cells, and *Foxj1*^+^ ependymal cells; UMAP: uniform manifold approximation and projection.

***Additional Figure 5:*** *Single-cell RNA sequencing showing gene expression profiles of five subpopulations of EPE cells.*

Additional Figure 5 Single-cell RNA sequencing showing gene expression profiles of five subpopulations of EPE cells.
(A) Layers of ependymal cells were categorized into five subgroups using a serial depletion method based on five representative EPE subtype-specific marker genes: *Prom1, Sox2, Vim, Pdgfrb*, and *Flt1*. (B) 97.7% of ependymal cells were defined by *Prom1*^+^, *Prom1*^-^/*Sox2*^+^, *Prom1*^-^/*Sox2*^-^/*Vim*^+^, *Prom1*^-^/*Sox2*^-^/*Vim*^-^/*Pdgfrb*^+^, and *Prom1*^-^/*Sox2*^-^ /*Vim*^-^/*Pdgfrb*^-^/*Flt1*^+^ subtypes. (C) The proportion of cells expressing different genes and their subgroup composition. Orange bars represent the proportion of cells expressing endothelial-like cell-specific genes (*Prom1, Pecam1, Flt1, Nes*, and *Notch1*), pericyte-like cell-specific genes (*Pdgfrb*), *Foxj1*^+^ ependymal cell-specific genes (*Sox2, Foxj1, S100b*, and *Acta2*), and other ependymal-related genes (*Vim, Vps35, Ccn1, Vcam1, Gfap*, and *Egfr*). Colored bars represent the subgroup composition of cells expressing these genes. (D) Pie charts show proportions of predominant gene expression. The top pie chart corresponds to the proportion of cells in the five subpopulations of A. The five pie charts at the bottom correspond to gene expression proportions of *Prom1* (gray), *Sox2* (green), *Pdgfrb* (red), *Flt1* (purple), and *Vim* (blue). Groups with cells < 0.1% are not shown. EPE cells: endothelial-like cells, pericyte-like cells, and *Foxj1*^+^ ependymal cells.

***Additional Figure 6:*** *Distribution of EPE cells co-expressing Prom1, Sox2, Vim, Pdgfrb, and Flt1.*

Additional Figure 6 Distribution of EPE cells co-expressing *Prom1, Sox2, Vim, Pdgfrb*, and *Flt1*.
(A) 97.7% of ependymal cells were defined by *Prom1*^+^, *Prom1*^-^/*Sox2*^+^, *Prom1*^-^/*Sox2*^-^/*Flt1*^+^, *Prom1*^-^/*Sox2*^-^/*Flt1*^-^/*Pdgfrb*^+^, and *Prom1*^-^/*Sox2*^-^/*Flt1*^-^/*Pdgfrb*^-^/*Vim*^+^ subtypes. The bottom section shows the proportion of cells expressing *Prom1, Sox2, Pdgfrb, Flt1*, and *Vim*. (B) 97.7% of ependymal cells were defined by *Prom1*^+^, *Prom1*^-^/*Flt1*^+^, *Prom1*^-^/*Flt1*^-^/*Sox2*^+^, *Prom1*^-^/*Flt1*^-^/*Sox2*^-^/*Pdgfrb*^+^, and *Prom1*^-^/*Flt1*^-^/*Sox2*^-^/*Pdgfrb*^-^/*Vim*^+^ subtypes. The bottom section shows the proportion of cells expressing *Prom1, Flt1, Sox2, Pdgfrb*, and *Vim*. (C) 97.7% of ependymal cells were defined by *Prom1*^+^, *Prom1*^-^/*Flt1*^+^, *Prom1*^-^/*Flt1*^-^/*Pdgfrb*^+^, *Prom1*^-^/*Flt1*^-^/*Pdgfrb*^-^/*Sox2*^+^, and *Prom1*^-^/*Flt1*^-^/*Pdgfrb*^-^/*Sox2*^-^/*Vim*^+^ subtypes. The bottom section shows the proportion of cells expressing *Prom1, Flt1, Pdgfrb, Sox2*, and *Vim*. EPE cells: endothelial-like cells, pericyte-like cells, and *Foxj1+* ependymal cells.

***Additional Figure 7:*** *Whole-mount immunocytochemical staining of the cellular composition of ependyma en face preparations.*

Additional Figure 7 Whole-mount immunocytochemical staining of the cellular composition of ependyma en face preparations.
(A) Proportions of ependymal cell subpopulations calculated using the Average 1 and Average 2 methods from whole-mount immunocytochemical staining. Average 1: Positive rates were added and mean values calculated. Average 2: Mean values of the first three layers of positive rates were added to merge positive rates to obtain a final mean value. (B) The proportion of ependymal cell subpopulations quantified using scRNA-seq. (C) Co-labeling of LECTIN, GFAP, EGFR (Cyanine Cy™3, red), CD133 (Alexa Fluor®647, white), and SOX2 (Alexa Fluor®488, green). Labeling of VIM (Cyanine Cy™3, red), CD133, SOX2, PDGFRβ (Alexa Fluor®647, white), and FLT1 (Alexa Fluor®488, green). White dotted circles represent cells expressing LECTIN, GFAP, EGFR or VIM; and yellow solid circles represent cells not expressing LECTIN, GFAP, EGFR or VIM. Scale bars: 10 μm. (D) The proportion of CD133^+^, CD133^-^/SOX2^+^, CD133^-^/SOX2^-^/VIM^+^, CD133^-^/SOX2^-^/VIM^-^/PDGFRβ^+^, and CD133^-^/SOX2^-^ /VIM^-^/PDGFR`^-^/FLT1^+^ cells in EPE cells measured by the Average 1 method from whole-mount immunocytochemical staining and single-cell sequencing. (E) The proportion of CD133^+^, CD133^-^/SOX2^+^, CD133^-^ /SOX2^-^VIM^+^, CD133^-^/SOX2^-^/VIM^-^/PDGFRβ^+^, and CD133^-^/SOX2^-^/VIM^-^/PDGFRβ^-^/FLT1^+^ cells in EPE cells measured by the Added method from whole-mount immunocytochemical staining and single-cell sequencing. (F) The proportion of CD133^+^, CD133^-^/SOX2^+^, CD133^-^/SOX2^-^/VIM+, CD133^-^/SOX2^-^/VIM^-^/PDGFRβ^+^, and CD133^-^ /SOX2^-^/VIM^-^/PDGFRβ^-^/FLT1^+^ cells in EPE cells measured by the merged method from whole-mount immunocytochemical staining and single-cell sequencing. EPE cells: endothelial-like cells, pericyte-like cells, and Foxj1+ ependymal cells.

***Additional Figure 8:*** *Added and merged image methods to calculate mean values.*

Additional Figure 8 Added and merged image methods to calculate mean values.
(A) Ratios to total nuclei (in ependymal surface) of CD133+ cells expressing SOX2, VIM, PDGFRβ, and FLT1 calculated using Added and Merged methods of immunocytochemical stained images. (B) Ratios to total nuclei (in ependymal surface) of CD133+ cells expressing CD31, NOTCH1, NESTIN, FOXJ1, S100B, ACTA2, and VPS35 calculated using Added and Merged methods of immunocytochemical stained images. (C) Ratios to total nuclei (in ependymal surface) of CD133+ cells expressing FLT1, CD31, NESTIN, SOX2, S100B, and PDGFRβ calculated using Added and Merged methods of immunocytochemical stained images. (D) Ratios to total nuclei (in ependymal surface) of FLT1, CD31, NESTIN, SOX2, and PDGFRβ co-labeling in CD133+ and CD133- samples calculated using Added and Merged methods of immunocytochemical stained images. (E) Merged images of whole-mount staining viewed from the ventricular surface. Stained using acetylated tubulin (Alexa Fluor®647, white) and nuclei (DAPI, blue). Scale bars: 10 μm. (F) Merged images of whole-mount staining viewed from the ventricular surface. Stained using gamma-tubulin (Alexa Fluor®647, white) and nuclei (DAPI, blue). Scale bars: 10 μm. DAPI: 4’,6- Diamidino-2-phenylindole dihydrochloride.

***Additional Figure 9:*** *Cellular view of protein expression of ependymal cells in en face preparation.*

Additional Figure 9 Cellular view of protein expression of ependymal cells in en face preparation.
(A) Schematic diagram illustrating en face ependymal cell composition from a coronal view. (B) Schematic diagram illustrating a cellular view of the major genes expressed in different ependymal cell types. (C) Schematic diagram illustrating a cellular view of the spatial location of protein expression in ependymal cells. Ependymal cells can be divided into five layers. Cilia Layer: ependymal cilia in direct contact with cerebrospinal fluid; Apical Layer: cilia root is connected to the cell membrane; Nuclei Layer: ependymal cell nuclei are located, as are tight connections between ependymal cells; Basal Layer: bottom layer of ependymal cells close to brain parenchyma, from a ventricular view; and Subependymal Layer: highly enriched in proliferating cells and neuroblasts. CSF: Cerebrospinal fluid.

***Additional Figure 10:*** *Expression profiles of Prom1*^*+*^ *and Prom1*^*–*^ *EPE cells in the spinal cord.*

Additional Figure 10 Expression profiles of *Prom1^+^* and *Prom1^-^* EPE cells in the spinal cord.
(A, B) Expression profile of *Prom1^+^* (A) and *Prom1^-^* (B) EPE cells in our previously published scRNA-seq data from adult mouse spinal cord with and without injury. The upper section shows the number of endothelial-like cells, pericyte-like cells, and *Foxj1^+^* ependymal cells. The lower sections show specific genes in these three clusters. EPE cells: Endothelial-like cells, pericyte-like cells, and *Foxj1^+^* ependymal cells.

***Additional Figure 11:*** *Expression profiles of EPE cells in the forebrain and spinal cord.*

Additional Figure 11 Expression profiles of EPE cells in the forebrain and spinal cord.
(A, C) Expression profile of forebrain (A) and spinal cord (B) in EPE cells. The upper section shows the number of endothelial-like cells, pericyte-like cells, and *Foxj1^+^* ependymal cells. The lower section shows specific genes in these three clusters. (C) UMAP of endothelial–pericyte-like cells (*Pdgfrb*^+^/*Foxj1*^+^) and *Foxj1*^+^ endothelial-like cells (*Foxj1*^+^/*Flt1*^+^). EPE cells: Endothelial-like cells, pericyte-like cells, and *Foxj1+* ependymal cells; UMAP: uniform manifold approximation and projection.

***Additional Figure 12:*** *Prom1 expression profiles in EPE cells.*

Additional Figure 12 *Prom1* expression profiles in EPE cells.
(A) UMAP of EPE subtypes, (B) UMAP of *Foxj1^+^* ependymal cells, (C) UMAP of pericyte-like cells, and (D) UMAP of endothelial-like cells. In all, red dots represent *Prom1^+^* cells and blue dots represent *Prom1^-^* cells. EPE cells: Endothelial-like cells, pericyte-like cells, and *Foxj1^+^* ependymal cells; UMAP: uniform manifold approximation and projection.

***Additional Table 1:*** *Information on antibodies.*

***Additional Table 2:*** *The top 50 genes highly expressed in each cell subtype.*

Additional Table 2 The top 50 genes highly expressed in each cell subtype

## Data Availability

*Single cell transcriptome sequencing data can be downloaded from the figshare website. Download link: https://figshare.com/s/0ceb6a946d996b8f6f26*.
