# Supplementary figures and images for: Heterogeneity of the adult mammalian forebrain neurogenic ependyma: A comprehensive cellular map
Source: Neural Regen Res. 2025 Apr 29;21(6):2448–56. doi: 10.4103/NRR.NRR-D-24-00789 (PMC13211782; doi:10.4103/NRR.NRR-D-24-00789)

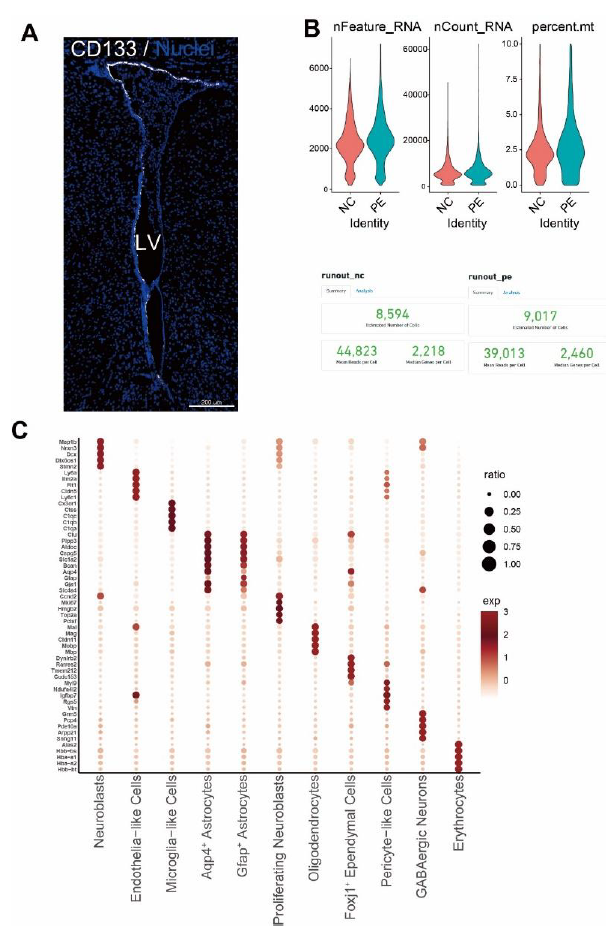

Supplement: Supplementary file 1 [file NRR-21-2448_Suppl1.tif]

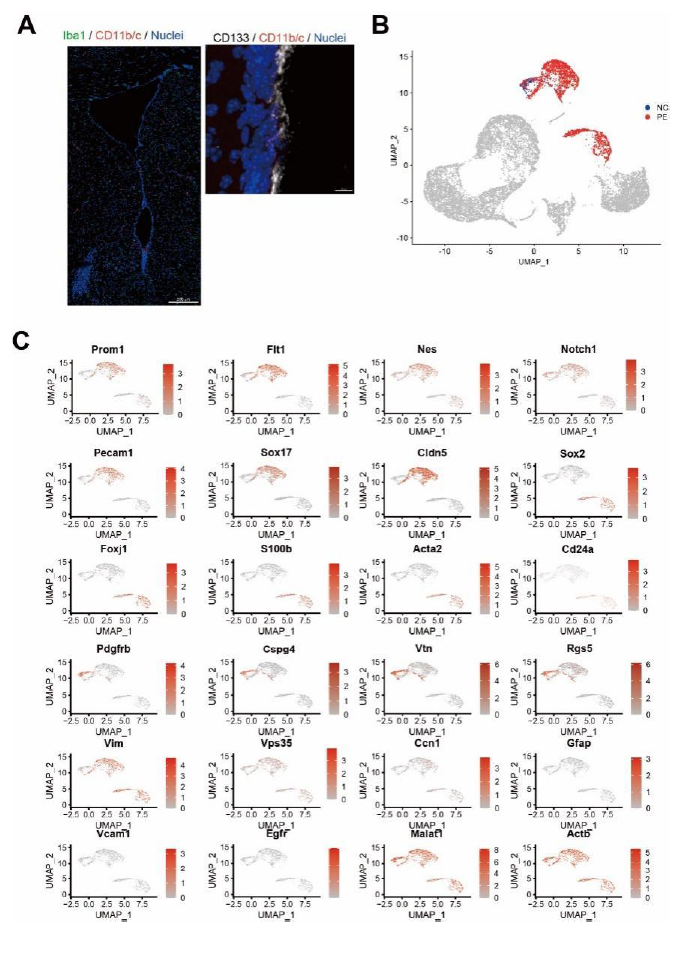

Supplement: Supplementary file 3 [file NRR-21-2448_Suppl2.tif]

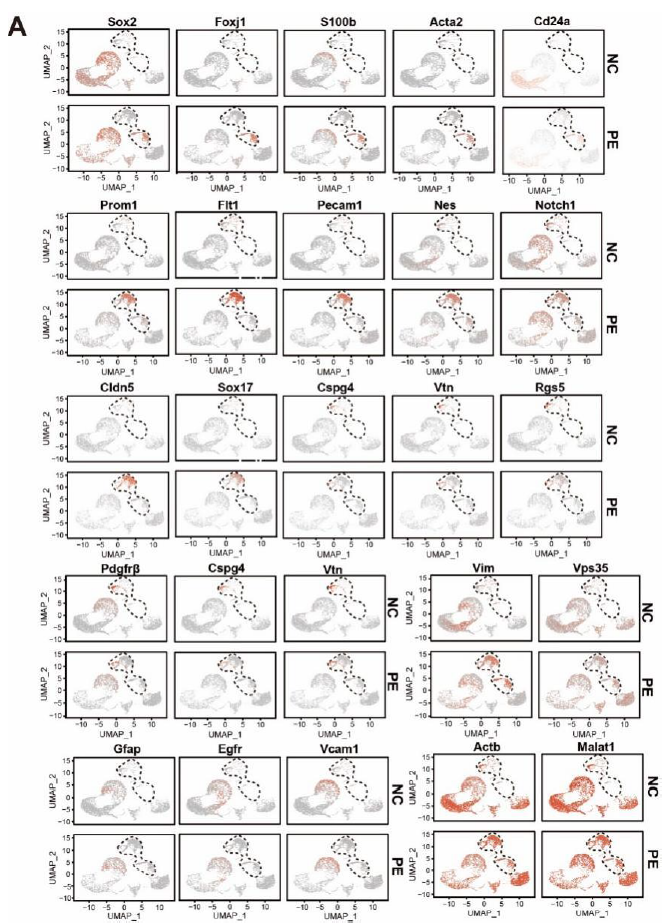

Supplement: Supplementary file 4 [file NRR-21-2448_Suppl3.tif]

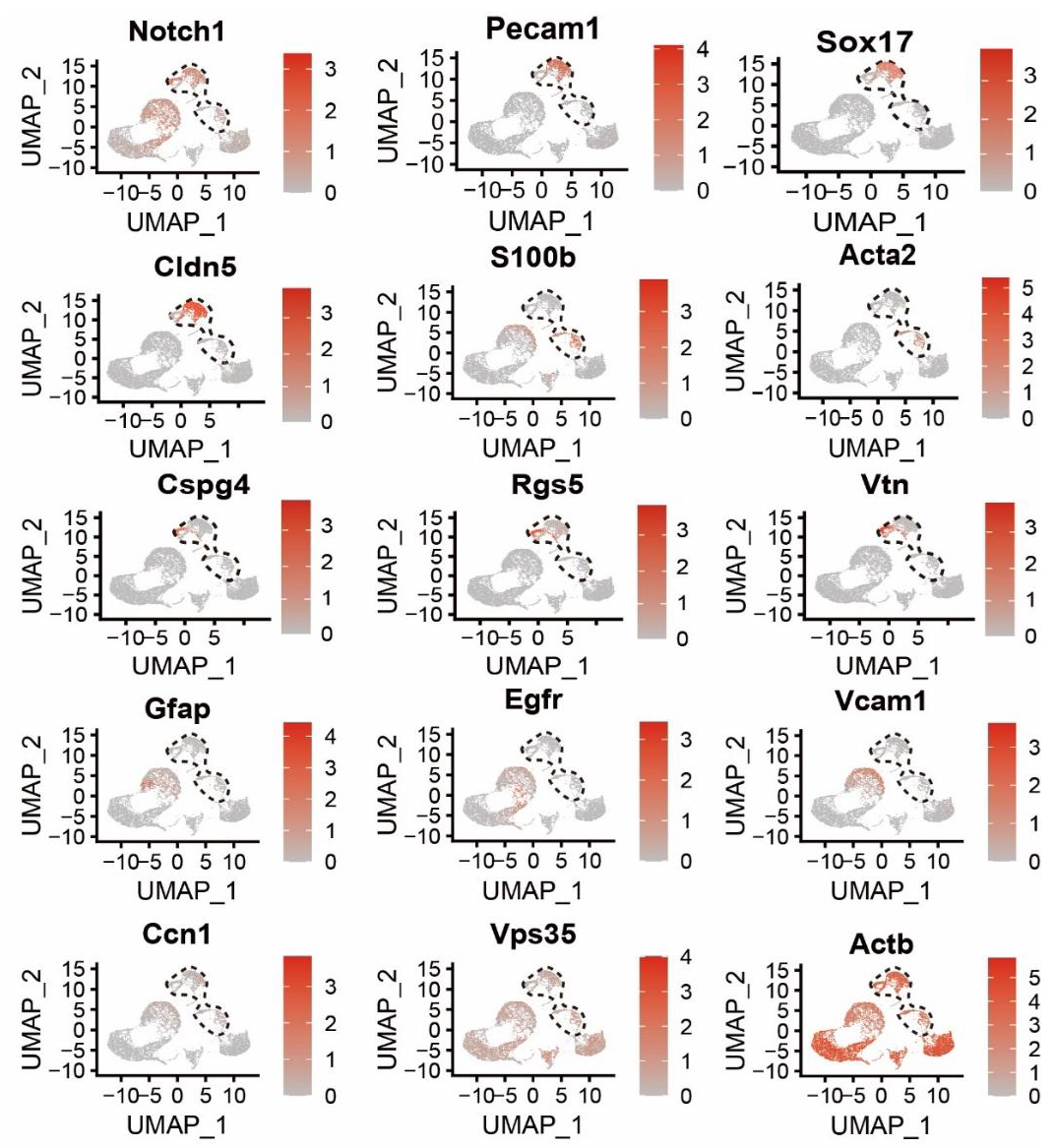

Supplement: Supplementary file 5 [file NRR-21-2448_Suppl4.tif]

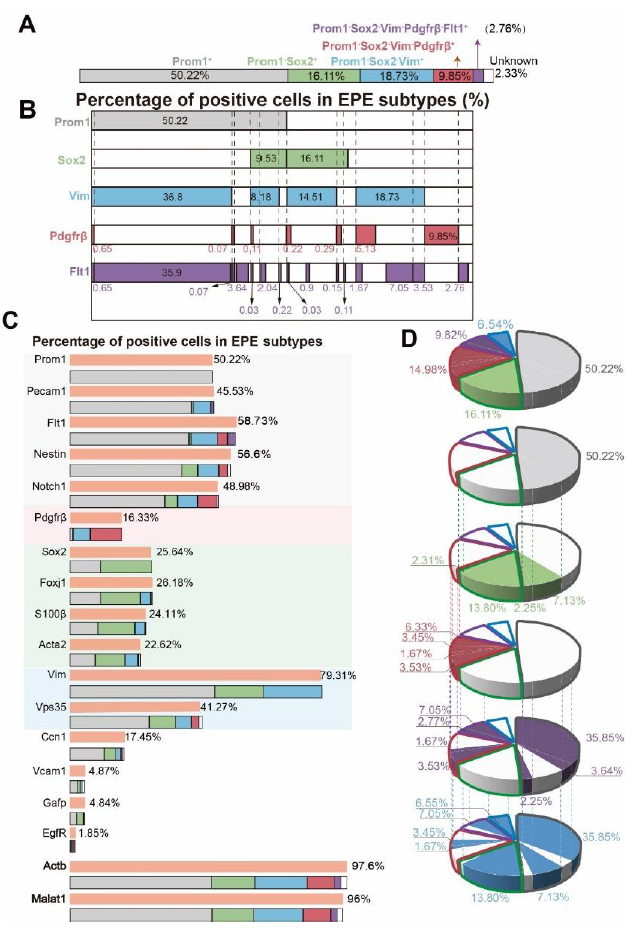

Supplement: Supplementary file 6 [file NRR-21-2448_Suppl5.tif]

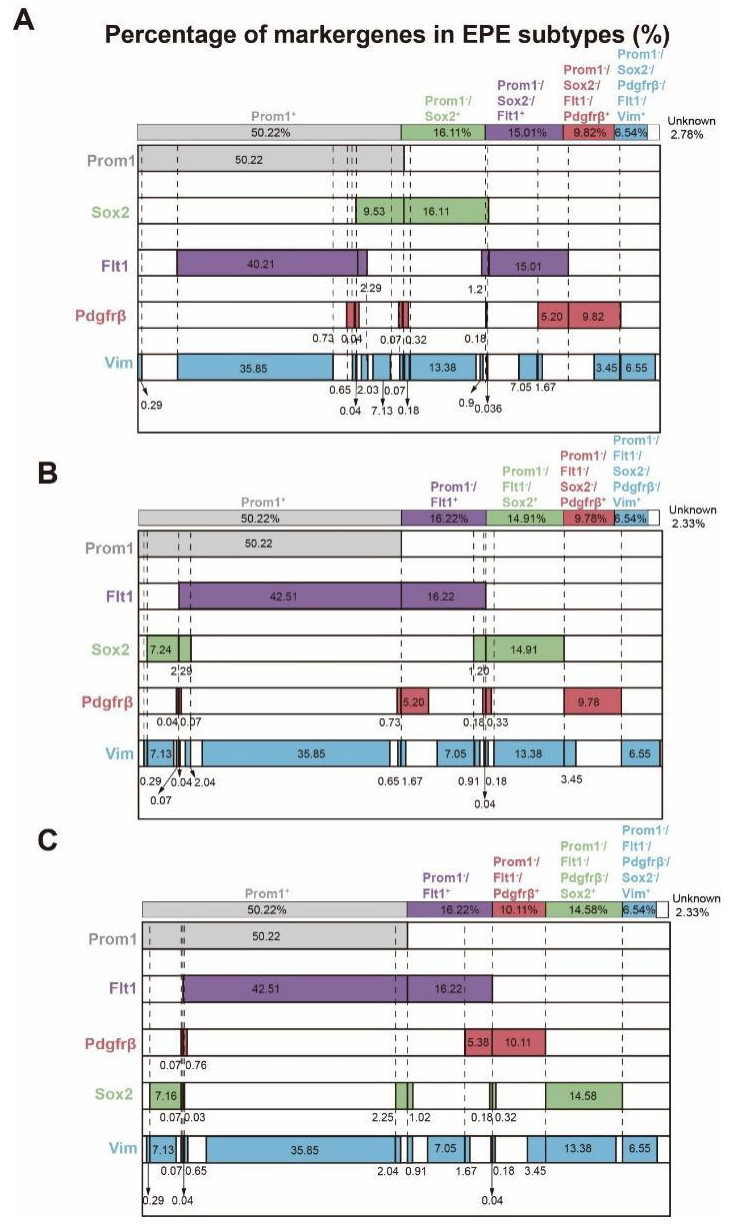

Supplement: Supplementary file 7 [file NRR-21-2448_Suppl6.tif]

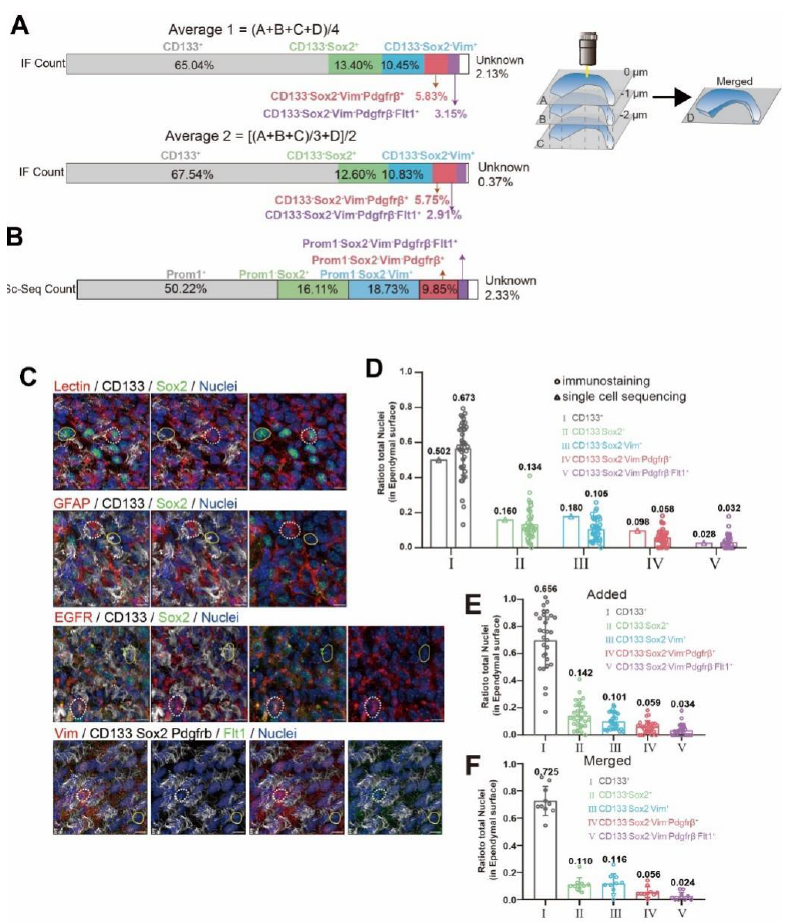

Supplement: Supplementary file 8 [file NRR-21-2448_Suppl7.tif]

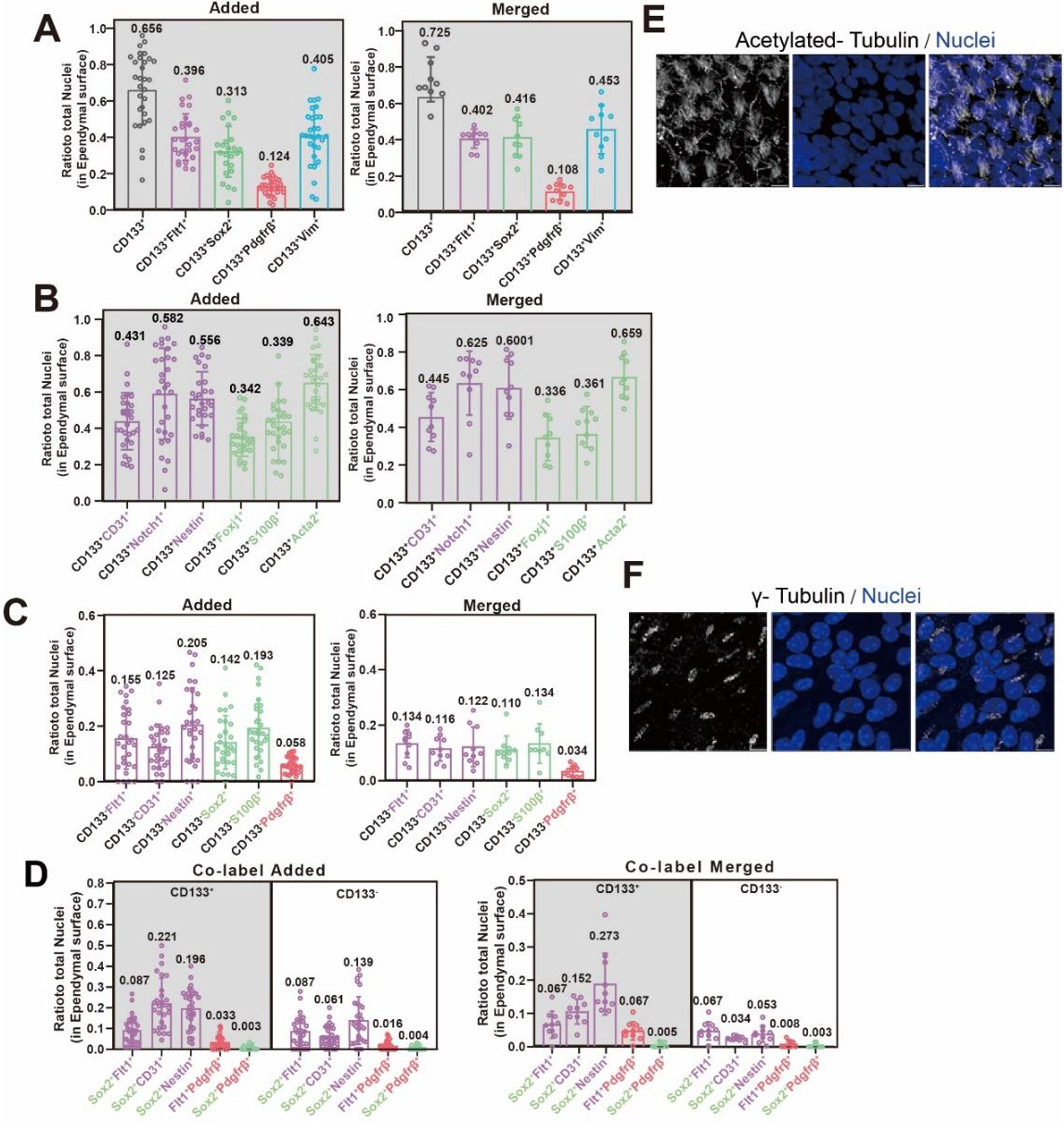

Supplement: Supplementary file 9 [file NRR-21-2448_Suppl8.tif]

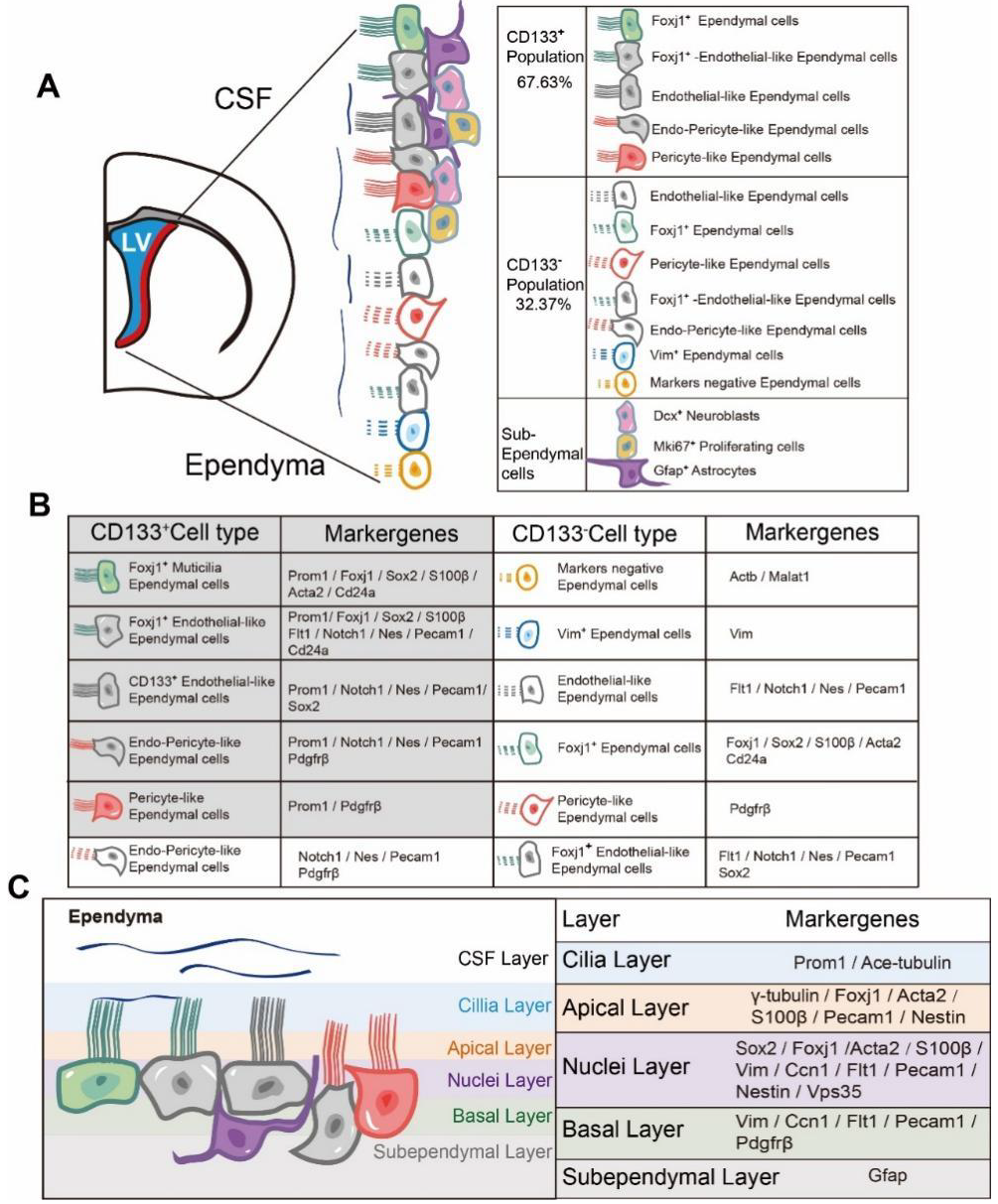

Supplement: Supplementary file 10 [file NRR-21-2448_Suppl9.tif]

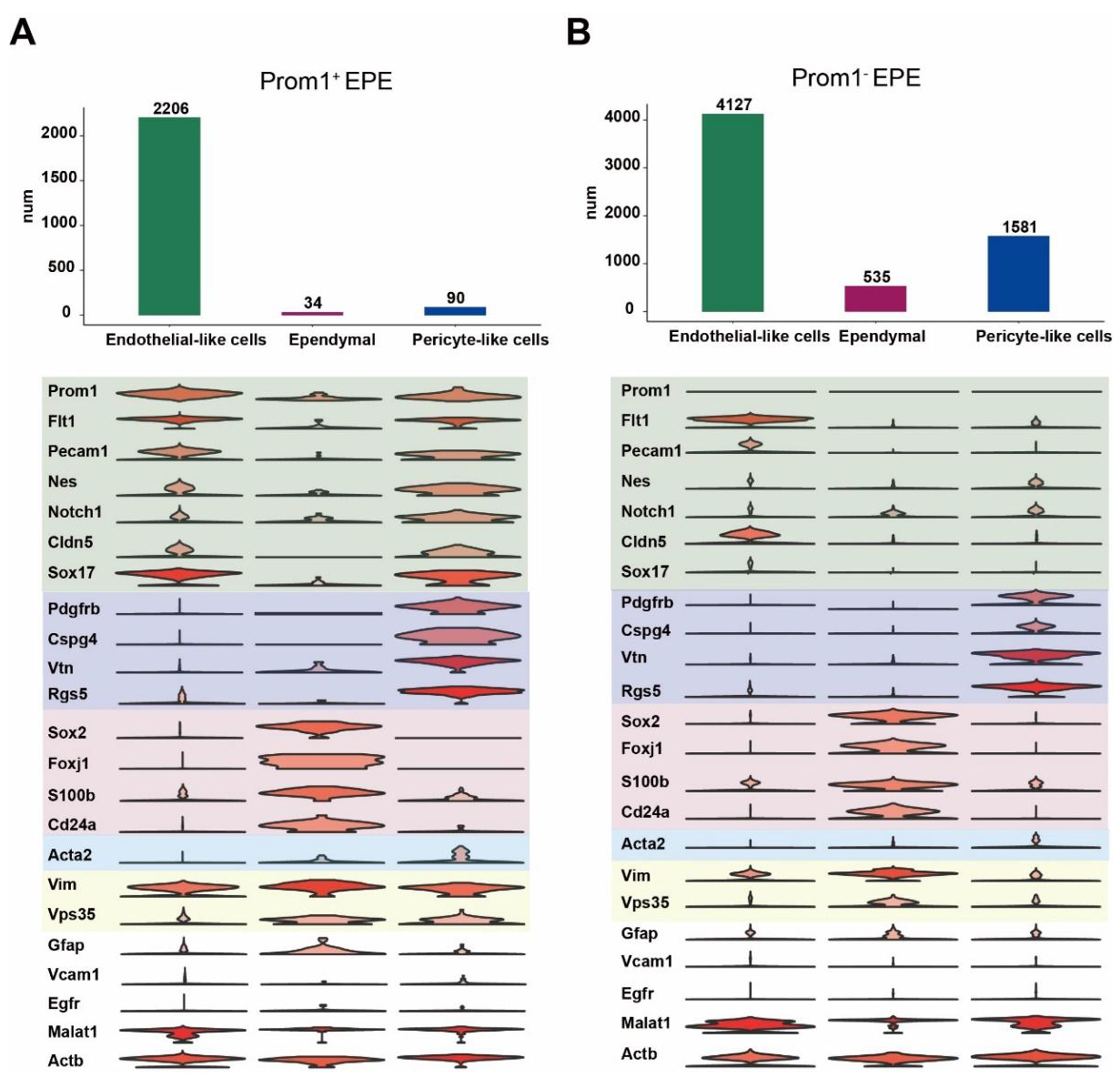

Supplement: Supplementary file 11 [file NRR-21-2448_Suppl10.tif]

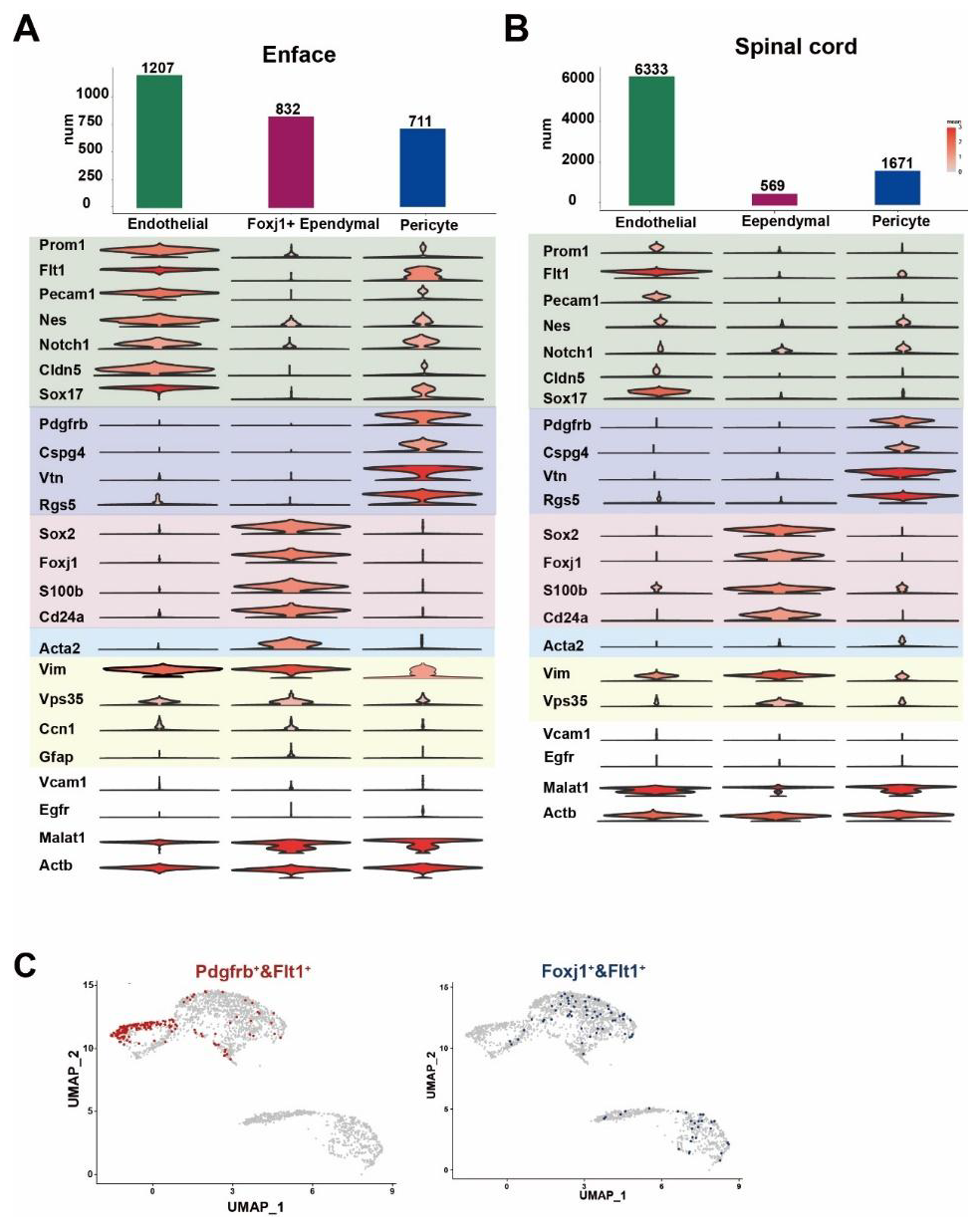

Supplement: Supplementary file 12 [file NRR-21-2448_Suppl11.tif]

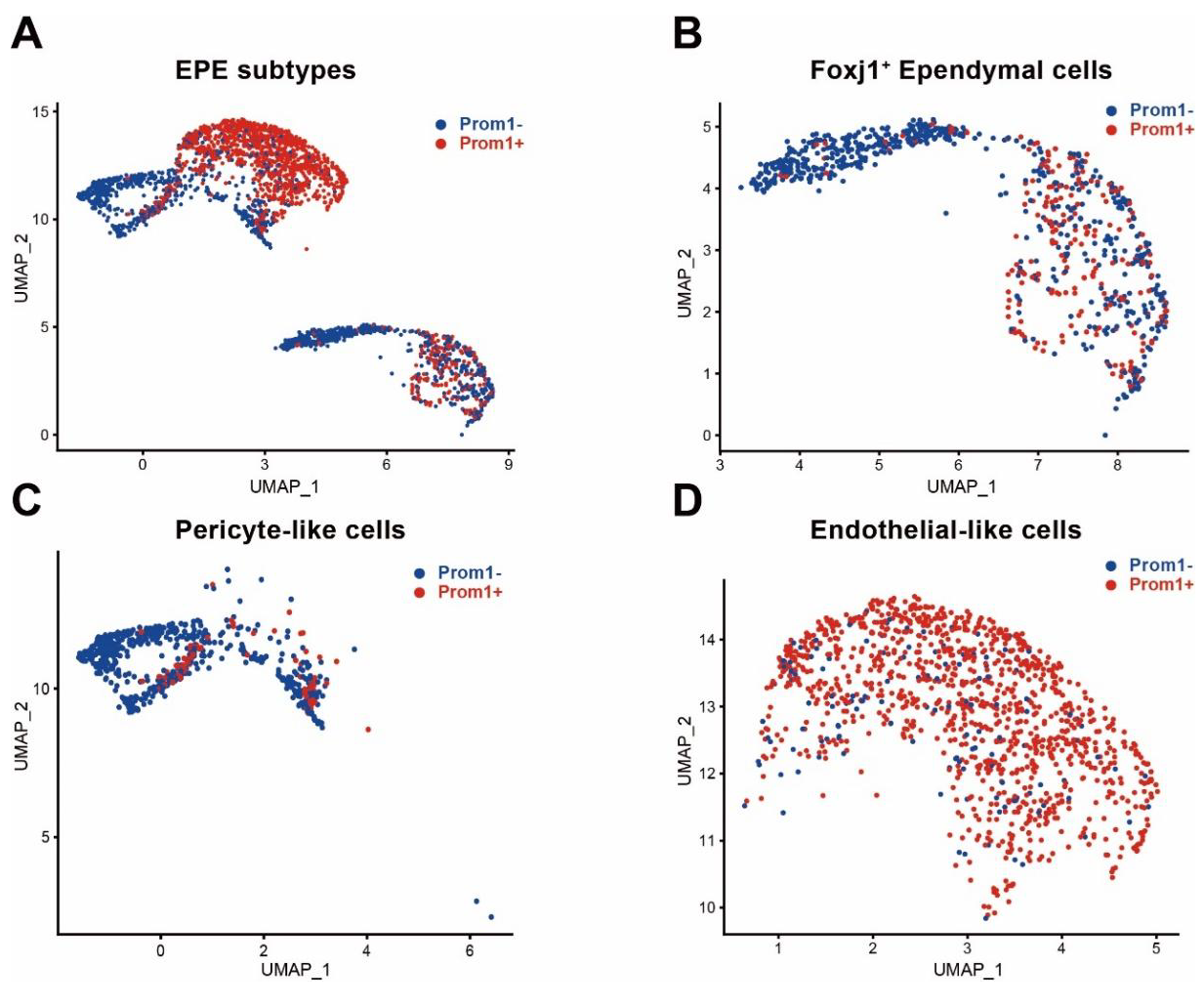

Supplement: Supplementary file 13 [file NRR-21-2448_Suppl12.tif]
