# Supplementary material for: Heterogeneity of the adult mammalian forebrain neurogenic ependyma: A comprehensive cellular map
Source: Neural Regen Res. 2025 Apr 29;21(6):2448–56. doi: 10.4103/NRR.NRR-D-24-00789 (PMC13211782; doi:10.4103/NRR.NRR-D-24-00789)
Supplement: Supplementary file 2 [file NRR-21-2448_Suppl1.pdf]

Additional Table 2: The top 50 genes highly expressed in each cell subtype

myAUC	avg_diff	power	avg_log2FC	pct.1	pct.2	cluster	cell type	gene
1	0.97	2.3452347	0.94	1.5398488	0.977	0.279	Neuroblast	*Map1b*
2	0.964	2.208886	0.928	1.5871405	0.961	0.107	Neuroblast	*Dcx*
3	0.964	2.1578941	0.928	1.5652379	0.977	0.156	Neuroblast	*Nrxn3*
4	0.956	2.2808599	0.912	1.6282523	0.955	0.09	Neuroblast	*Dlx6os1*
5	0.955	1.9626319	0.91	1.2761114	0.961	0.401	Neuroblast	*Rtn1*
6	0.954	2.0668482	0.908	1.3236219	0.972	0.39	Neuroblast	*Sox4*
7	0.953	1.6489531	0.906	1.0226644	0.987	0.559	Neuroblast	*Nfib*
8	0.953	2.489705	0.906	1.6965814	0.94	0.122	Neuroblast	*Stmn2*
9	0.944	1.8354443	0.888	1.2048632	0.976	0.388	Neuroblast	*Meis2*
10	0.942	1.8903387	0.884	1.3902052	0.933	0.128	Neuroblast	*Celf4*
11	0.941	2.1663005	0.882	1.2554372	0.945	0.464	Neuroblast	*Tubb2b*
12	0.937	1.6230618	0.874	1.1281057	0.966	0.405	Neuroblast	*Ncam1*
13	0.935	1.8718675	0.87	1.4650745	0.978	0.237	Neuroblast	*Sox11*
14	0.933	1.9654125	0.866	1.447295	0.919	0.102	Neuroblast	*Igfbpl1*
15	0.933	1.6143519	0.866	1.0370885	0.974	0.498	Neuroblast	*Pbx1*
16	0.932	1.9699381	0.864	1.3799455	0.907	0.163	Neuroblast	*Nrep*
17	0.932	2.0475077	0.864	1.3372169	0.922	0.272	Neuroblast	*Zfp704*
18	0.931	1.5624011	0.862	1.010315	0.954	0.526	Neuroblast	*Ttc3*
19	0.931	1.7207981	0.862	0.8040673	0.977	0.849	Neuroblast	*Tuba1a*
20	0.93	2.2452857	0.86	1.5390136	0.911	0.151	Neuroblast	*Tubb3*
21	0.93	2.1031825	0.86	1.293344	0.936	0.466	Neuroblast	*Tmsb10*
22	0.927	1.8743816	0.854	1.3795515	0.913	0.138	Neuroblast	*Dpysl3*
23	0.917	1.7330845	0.834	1.1532857	0.96	0.557	Neuroblast	*Stmn1*
24	0.911	1.4321834	0.822	0.9813692	0.973	0.455	Neuroblast	*Auts2*
25	0.91	1.6504214	0.82	1.2288045	0.885	0.179	Neuroblast	*Stmn3*
26	0.907	1.450857	0.814	1.0388668	0.943	0.409	Neuroblast	*Basp1*
27	0.907	1.6080901	0.814	1.1758268	0.911	0.292	Neuroblast	*Marcksl1*
28	0.905	1.3215801	0.81	0.5762946	0.957	0.894	Neuroblast	*H3f3a*
29	0.901	1.5573561	0.802	1.1898129	0.86	0.13	Neuroblast	*Nsg2*
30	0.901	1.600717	0.802	1.2537343	0.878	0.105	Neuroblast	*Dlx1*
31	0.899	1.4724843	0.798	1.06539	0.907	0.344	Neuroblast	*Jpt1*
32	0.893	1.3737601	0.786	0.9820657	0.919	0.395	Neuroblast	*Ptprs*
33	0.893	1.5969719	0.786	1.0393261	0.935	0.59	Neuroblast	*Tubb5*
34	0.891	1.6568178	0.782	1.0891625	0.906	0.416	Neuroblast	*Pfn2*
35	0.89	1.2154154	0.78	0.7958993	0.966	0.606	Neuroblast	*Ccdc88a*
36	0.889	1.4348484	0.778	0.978015	0.919	0.502	Neuroblast	*Ppp1r14b*
37	0.877	1.4048647	0.754	1.0316354	0.855	0.268	Neuroblast	*Gng2*
38	0.877	1.2125853	0.754	0.8069643	0.917	0.589	Neuroblast	*Atpif1*
39	0.877	1.1548295	0.754	1.0665043	0.949	0.429	Neuroblast	*Nnat*
40	0.873	1.7109066	0.746	1.2081213	0.823	0.155	Neuroblast	*Shtn1*
41	0.871	1.4729288	0.742	1.0697079	0.821	0.205	Neuroblast	*Serf1*
42	0.87	1.1714763	0.74	0.7461667	0.931	0.66	Neuroblast	*Prdx2*
43	0.869	1.3492381	0.738	1.0177173	0.823	0.195	Neuroblast	*Rbfox2*
44	0.869	1.276945	0.738	1.0303454	0.886	0.232	Neuroblast	*Elavl3*
45	0.867	1.303008	0.734	0.987568	0.861	0.279	Neuroblast	*Gm1673*
46	0.866	1.5903406	0.732	1.1663614	0.755	0.045	Neuroblast	*Islr2*
47	0.866	1.3870026	0.732	1.0775976	0.795	0.107	Neuroblast	*Crmp1*
48	0.866	1.3708933	0.732	1.0505432	0.792	0.137	Neuroblast	*Mllt11*
49	0.862	1.3089156	0.724	1.0127695	0.812	0.174	Neuroblast	*Dbn1*
50	0.862	1.3158816	0.724	1.0750756	0.854	0.183	Neuroblast	*Cd24a*
51	0.993	3.2453613	0.986	2.0921413	0.999	0.059	Endothelia-like_Cell	*Itm2a*

52	0.993	3.4027443	0.986	2.1628638	0.998	0.046	Endothelia-like_Cell	*Cldn5*
53	0.993	3.2841848	0.986	2.1300672	1	0.056	Endothelia-like_Cell	*Flt1*
54	0.992	3.0722809	0.984	1.3931878	1	0.762	Endothelia-like_Cell	*Bsg*
55	0.992	2.8740953	0.984	1.95254	0.998	0.033	Endothelia-like_Cell	*Slco1a4*
56	0.991	3.3184673	0.982	2.175874	0.999	0.062	Endothelia-like_Cell	*Ly6c1*
57	0.991	2.9332566	0.982	1.8802043	0.997	0.144	Endothelia-like_Cell	*Spock2*
58	0.989	2.987824	0.978	2.0189588	0.997	0.044	Endothelia-like_Cell	*Ly6a*
59	0.989	2.6316045	0.978	1.7186627	0.997	0.22	Endothelia-like_Cell	*Slc2a1*
60	0.988	2.238466	0.976	1.2919529	0.998	0.532	Endothelia-like_Cell	*Sptbn1*
61	0.988	2.730809	0.976	1.8691494	0.988	0.027	Endothelia-like_Cell	*Ptprb*
62	0.987	2.6104431	0.974	1.7942612	0.993	0.09	Endothelia-like_Cell	*Ramp2*
63	0.985	2.4163426	0.97	1.6957768	0.991	0.096	Endothelia-like_Cell	*Slc9a3r2*
64	0.984	2.2789821	0.968	1.6709909	0.983	0.018	Endothelia-like_Cell	*Adgrl4*
65	0.984	2.5957571	0.968	1.7860386	1	0.172	Endothelia-like_Cell	*Pltp*
66	0.983	2.1738894	0.966	1.3335626	0.996	0.462	Endothelia-like_Cell	*Tsc22d1*
67	0.983	2.2147054	0.966	1.5985582	0.985	0.095	Endothelia-like_Cell	*Abcg2*
68	0.983	2.3437758	0.966	1.7014452	0.98	0.02	Endothelia-like_Cell	*Abcb1a*
69	0.983	2.3233876	0.966	1.6754064	0.98	0.036	Endothelia-like_Cell	*Egfl7*
70	0.982	2.3455112	0.964	1.6994615	0.981	0.034	Endothelia-like_Cell	*Adgrf5*
71	0.978	2.2843548	0.956	1.6594215	0.973	0.048	Endothelia-like_Cell	*Jcad*
72	0.977	2.1242537	0.954	1.5378972	0.976	0.114	Endothelia-like_Cell	*Sgms1*
73	0.975	2.5781857	0.95	1.7738913	0.967	0.029	Endothelia-like_Cell	*Pglyrp1*
74	0.975	2.2860196	0.95	1.6353207	0.966	0.051	Endothelia-like_Cell	*Vwa1*
75	0.973	2.0372792	0.946	1.4551896	0.991	0.213	Endothelia-like_Cell	*Slco1c1*
76	0.972	2.2871313	0.944	1.5716445	0.976	0.144	Endothelia-like_Cell	*Id1*
77	0.971	1.9868325	0.942	1.4750263	0.986	0.158	Endothelia-like_Cell	*Epas1*
78	0.968	1.8899744	0.936	1.3692856	0.997	0.285	Endothelia-like_Cell	*Ly6e*
79	0.967	2.1597317	0.934	1.5327742	0.96	0.109	Endothelia-like_Cell	*Kitl*

80	0.967	1.9894327	0.934	1.5100468	0.956	0.035	Endothelia -like_Cell	*Esam*
81	0.966	2.0299974	0.932	1.4775917	0.968	0.127	Endothelia -like_Cell	*Ablim1*
82	0.963	2.2096818	0.926	1.6688975	0.98	0.095	Endothelia -like_Cell	*Hspb1*
83	0.961	1.8781585	0.922	1.4472771	0.94	0.034	Endothelia -like_Cell	*Pecam1*
84	0.961	1.8926845	0.922	1.4344396	0.954	0.076	Endothelia -like_Cell	*Cd34*
85	0.96	2.0644737	0.92	1.7274601	0.993	0.084	Endothelia -like_Cell	*Igfbp7*
86	0.958	2.0379988	0.916	1.4659139	0.949	0.101	Endothelia -like_Cell	*Ccdc141*
87	0.955	1.4157995	0.91	0.813758	0.991	0.747	Endothelia -like_Cell	*Myl6*
88	0.955	1.8994874	0.91	1.4093891	0.962	0.175	Endothelia -like_Cell	*Utrn*
89	0.953	1.780723	0.906	1.3834705	0.923	0.035	Endothelia -like_Cell	*Kank3*
90	0.951	1.866627	0.902	1.4338899	0.925	0.027	Endothelia -like_Cell	*Emcn*
91	0.951	1.8648193	0.902	1.4272546	0.918	0.018	Endothelia -like_Cell	*Nostrin*
92	0.95	1.8602968	0.9	1.4028907	0.934	0.086	Endothelia -like_Cell	*Pakap.1*
93	0.949	1.8212639	0.898	1.398501	0.907	0.01	Endothelia -like_Cell	*Cyyr1*
94	0.949	2.0313922	0.898	1.4872339	0.916	0.033	Endothelia -like_Cell	*Sema3c*
95	0.947	1.929017	0.894	1.5624225	0.985	0.15	Endothelia -like_Cell	*Klf2*
96	0.945	1.7144091	0.89	1.3293784	0.942	0.126	Endothelia -like_Cell	*Srgn*
97	0.943	1.729543	0.886	1.2340146	0.942	0.247	Endothelia -like_Cell	*Slc39a10*
98	0.942	1.7701368	0.884	1.3723602	0.899	0.018	Endothelia -like_Cell	*Wfdc1*
99	0.942	3.086806	0.884	1.8735013	0.909	0.064	Endothelia -like_Cell	*Cxcl12*
100	0.942	1.7108882	0.884	1.3078953	0.925	0.101	Endothelia -like_Cell	*Eng*
101	0.989	4.1118216	0.978	2.2223248	0.98	0.117	Microglia	*C1qa*
102	0.988	3.41934	0.976	2.067205	0.978	0.055	Microglia	*Csf1r*
103	0.987	3.8391075	0.974	2.1773236	0.976	0.077	Microglia	*C1qc*
104	0.987	4.0034029	0.974	2.1874118	0.976	0.118	Microglia	*C1qb*
105	0.986	3.2186256	0.972	1.6089243	0.985	0.5	Microglia	*Ctsd*
106	0.985	3.9212319	0.97	2.0704691	0.977	0.22	Microglia	*Hexb*
107	0.985	3.8017899	0.97	2.1429537	0.973	0.085	Microglia	*Ctss*
108	0.979	3.5824617	0.958	2.0942713	0.96	0.048	Microglia	*Cx3cr1*
109	0.979	3.0489912	0.958	1.9411701	0.961	0.033	Microglia	*Laptm5*
110	0.975	3.0656585	0.95	1.9309957	0.953	0.041	Microglia	*Trem2*
111	0.975	2.7916584	0.95	1.8313864	0.953	0.027	Microglia	*Ly86*
112	0.974	2.9502795	0.948	1.8721931	0.951	0.029	Microglia	*Fcrls*
113	0.972	2.6818354	0.944	1.6356194	0.959	0.27	Microglia	*Lgmn*
114	0.972	3.0776772	0.944	1.926712	0.946	0.032	Microglia	*Selplg*

115	0.971	2.4609572	0.942	1.6806422	0.949	0.05	Microglia	*Vsir*
116	0.971	2.9048708	0.942	1.8746303	0.947	0.033	Microglia	*Fcer1g*
117	0.97	3.0718783	0.94	1.9176233	0.945	0.048	Microglia	*Tyrobp*
118	0.968	2.8595272	0.936	1.8436917	0.939	0.019	Microglia	*Siglech*
119	0.967	2.8001252	0.934	1.8159941	0.938	0.025	Microglia	*Gpr34*
120	0.966	3.1468152	0.932	1.925856	0.936	0.038	Microglia	*P2ry12*
121	0.964	2.1678254	0.928	1.5533791	0.937	0.052	Microglia	*Unc93b1*
122	0.962	2.0614917	0.924	0.8111919	0.999	0.973	Microglia	*Cst3*
123	0.96	2.2658899	0.92	1.5473607	0.939	0.092	Microglia	*Kctd12*
124	0.959	2.4600606	0.918	1.6485275	0.929	0.088	Microglia	*Rgs10*
125	0.959	2.6701276	0.918	1.749318	0.923	0.035	Microglia	*Olfml3*
126	0.959	2.0946747	0.918	1.3211077	0.953	0.375	Microglia	*Ctsz*
127	0.958	1.9707311	0.916	1.1575176	0.96	0.515	Microglia	*Ctsb*
128	0.956	2.0632473	0.912	1.3192667	0.958	0.401	Microglia	*Serinc3*
129	0.955	2.0872533	0.91	1.4544753	0.934	0.15	Microglia	*Itgb5*
130	0.955	2.6506657	0.91	1.7386811	0.915	0.02	Microglia	*Tmem119*
131	0.954	2.1629268	0.908	1.5246461	0.916	0.034	Microglia	*Hpgds*
132	0.954	2.2627822	0.908	1.5989199	0.911	0.014	Microglia	*Fcgr3*
133	0.95	1.9878634	0.9	1.4542476	0.904	0.016	Microglia	*Cyth4*
134	0.95	1.9728713	0.9	1.3706297	0.928	0.195	Microglia	*Grn*
135	0.949	2.1353088	0.898	1.4561211	0.922	0.145	Microglia	*Tgfbr1*
136	0.947	1.9961744	0.894	1.4452529	0.903	0.031	Microglia	*Fyb*
137	0.947	1.9216025	0.894	1.3532262	0.946	0.191	Microglia	*Mef2c*
138	0.947	2.0445089	0.894	1.4744899	0.902	0.04	Microglia	*Lpcat2*
139	0.946	2.1084399	0.892	1.4232742	0.94	0.252	Microglia	*B2m*
140	0.944	2.2157767	0.888	1.518366	0.902	0.047	Microglia	*Maf*
141	0.943	2.0755424	0.886	1.4918298	0.891	0.011	Microglia	*Mpeg1*
142	0.943	1.949957	0.886	1.4297184	0.888	0.011	Microglia	*Pld4*
143	0.943	2.1434008	0.886	1.5206879	0.89	0.013	Microglia	*Itgam*
144	0.943	1.8907171	0.886	1.387551	0.901	0.055	Microglia	*Lyn*
145	0.942	2.3900227	0.884	1.5195928	0.903	0.083	Microglia	*Mafb*
146	0.941	1.9852899	0.882	1.4315832	0.893	0.05	Microglia	*Lair1*
147	0.939	1.9819206	0.878	1.440885	0.884	0.017	Microglia	*Ptgs1*
148	0.935	1.8816049	0.87	1.2351155	0.932	0.341	Microglia	*Epb41l2*
149	0.935	1.8772757	0.87	1.403629	0.876	0.011	Microglia	*Cd53*
150	0.935	1.9195397	0.87	1.4200042	0.897	0.053	Microglia	*Cfh*
151	0.938	1.9442971	0.876	1.1102566	0.97	0.597	Gfap+_Astrocyte	*Cpe*
152	0.901	1.6770608	0.802	1.1684718	0.939	0.358	Gfap+_Astrocyte	*Ntrk2*
153	0.893	1.4444753	0.786	1.2897367	0.985	0.447	Gfap+_Astrocyte	*Slc1a2*
154	0.89	1.3066851	0.78	0.8680208	0.997	0.845	Gfap+_Astrocyte	*Apoe*
155	0.886	1.4967746	0.772	1.0012139	0.94	0.517	Gfap+_Astrocyte	*Prdx6*
156	0.884	1.5451246	0.768	1.2808469	0.928	0.237	Gfap+_Astrocyte	*Cspg5*
157	0.882	1.5245703	0.764	1.1270407	0.891	0.256	Gfap+_Astrocyte	*Dtna*
158	0.881	1.4470631	0.762	1.1298442	0.942	0.386	Gfap+_Astrocyte	*Acsl3*
159	0.878	1.6043435	0.756	1.2018776	0.957	0.416	Gfap+_Astrocyte	*Clu*

160	0.871	1.37291	0.742	0.9841849	0.909	0.44	Gfap+_Astrocyte	*Ndrg2*
161	0.871	1.6059492	0.742	1.2580259	0.9	0.278	Gfap+_Astrocyte	*Aldoc*
162	0.87	1.3156179	0.74	1.2024376	0.948	0.341	Gfap+_Astrocyte	*Plpp3*
163	0.861	1.1282904	0.722	0.6279004	0.975	0.81	Gfap+_Astrocyte	*Ckb*
164	0.861	1.6791121	0.722	1.1604032	0.829	0.217	Gfap+_Astrocyte	*Fxyd1*
165	0.855	1.249437	0.71	1.000517	0.926	0.37	Gfap+_Astrocyte	*Nrxn1*
166	0.849	1.086018	0.698	0.7547162	0.93	0.585	Gfap+_Astrocyte	*Gpm6b*
167	0.846	1.118072	0.692	0.9876819	0.928	0.411	Gfap+_Astrocyte	*Slc1a3*
168	0.845	1.1954005	0.69	0.8403895	0.968	0.675	Gfap+_Astrocyte	*Mt1*
169	0.845	0.939064	0.69	0.755587	0.946	0.736	Gfap+_Astrocyte	*Dbi*
170	0.844	1.2382186	0.688	0.9381746	0.89	0.373	Gfap+_Astrocyte	*Id4*
171	0.842	1.1609085	0.684	1.0296292	0.893	0.269	Gfap+_Astrocyte	*Ttyh1*
172	0.838	1.32614	0.676	0.9341539	0.835	0.351	Gfap+_Astrocyte	*Phgdh*
173	0.837	1.1193464	0.674	1.0202012	0.943	0.446	Gfap+_Astrocyte	*Mt3*
174	0.834	1.449443	0.668	1.0403205	0.783	0.219	Gfap+_Astrocyte	*Ddah1*
175	0.822	1.0293023	0.644	0.8474765	0.918	0.427	Gfap+_Astrocyte	*Ptprz1*
176	0.819	1.0417647	0.638	0.9685947	0.834	0.187	Gfap+_Astrocyte	*Ntsr2*
177	0.819	1.2322089	0.638	0.8902413	0.855	0.408	Gfap+_Astrocyte	*Gstm1*
178	0.819	0.9868144	0.638	0.9468013	0.837	0.19	Gfap+_Astrocyte	*Bcan*
179	0.816	0.9170964	0.632	0.9672517	0.872	0.26	Gfap+_Astrocyte	*Gja1*
180	0.814	1.1271983	0.628	0.8967714	0.811	0.241	Gfap+_Astrocyte	*Rorb*
181	0.808	1.126438	0.616	0.8911688	0.845	0.348	Gfap+_Astrocyte	*Mt2*
182	0.807	0.8563193	0.614	0.7840289	0.955	0.578	Gfap+_Astrocyte	*Ptn*
183	0.806	1.4992105	0.612	1.0349028	0.739	0.21	Gfap+_Astrocyte	*S100a6*
184	0.806	1.5188875	0.612	0.9945544	0.776	0.266	Gfap+_Astrocyte	*Igfbp5*
185	0.804	1.1459649	0.608	0.8996342	0.797	0.276	Gfap+_Astrocyte	*Sox9*
186	0.802	1.0825921	0.604	0.8252689	0.789	0.315	Gfap+_Astrocyte	*Asrgl1*
187	0.799	1.1227543	0.598	0.8581546	0.718	0.177	Gfap+_Astrocyte	*Sfxn5*

188	0.799	1.3715944	0.598	0.9539778	0.686	0.163	Gfap+_Astrocyte	*Fbxo2*
189	0.799	0.9843511	0.598	0.7737064	0.902	0.467	Gfap+_Astrocyte	*Dclk1*
190	0.795	1.1001138	0.59	0.8574605	0.751	0.209	Gfap+_Astrocyte	*Fam107a*
191	0.794	0.7353366	0.588	0.6350981	0.924	0.632	Gfap+_Astrocyte	*Scd2*
192	0.794	0.8922751	0.588	0.5299399	0.968	0.867	Gfap+_Astrocyte	*AY036118*
193	0.784	1.0571732	0.568	0.687955	0.836	0.492	Gfap+_Astrocyte	*Mir99ahg*
194	0.784	0.9342742	0.568	0.7169569	0.851	0.476	Gfap+_Astrocyte	*Prnp*
195	0.783	0.9522062	0.566	0.7938937	0.822	0.335	Gfap+_Astrocyte	*Tmem47*
196	0.78	0.9614416	0.56	0.6760406	0.807	0.491	Gfap+_Astrocyte	*Glud1*
197	0.78	1.507972	0.56	0.9972358	0.597	0.053	Gfap+_Astrocyte	*Thbs4*
198	0.776	0.8832407	0.552	0.5754713	0.876	0.613	Gfap+_Astrocyte	*Msi2*
199	0.775	1.0040839	0.55	0.7554092	0.71	0.252	Gfap+_Astrocyte	*Emp2*
200	0.775	0.9822844	0.55	0.7831625	0.71	0.193	Gfap+_Astrocyte	*Mlc1*
201	0.981	2.2502383	0.962	1.5352758	0.996	0.242	Aqp4+_Astrocyte	*Gja1*
202	0.981	2.2414092	0.962	1.5790175	0.989	0.148	Aqp4+_Astrocyte	*Slc4a4*
203	0.98	2.2221758	0.96	1.5001967	0.993	0.235	Aqp4+_Astrocyte	*Htra1*
204	0.98	2.2748574	0.96	1.5378124	1	0.331	Aqp4+_Astrocyte	*Plpp3*
205	0.977	2.3760494	0.954	1.5346707	1	0.442	Aqp4+_Astrocyte	*Slc1a2*
206	0.976	2.0662402	0.952	1.480553	0.991	0.194	Aqp4+_Astrocyte	*Gpr37l1*
207	0.975	2.0729978	0.95	1.3739782	0.999	0.4	Aqp4+_Astrocyte	*Slc1a3*
208	0.975	2.0595136	0.95	1.5070876	0.993	0.168	Aqp4+_Astrocyte	*Bcan*
209	0.971	1.9905962	0.942	1.4865856	0.993	0.165	Aqp4+_Astrocyte	*Ntsr2*
210	0.97	1.9054106	0.94	1.4069997	0.997	0.253	Aqp4+_Astrocyte	*Ttyh1*
211	0.969	2.0076971	0.938	1.4746601	0.978	0.133	Aqp4+_Astrocyte	*F3*
212	0.963	2.030182	0.926	1.4828018	0.97	0.134	Aqp4+_Astrocyte	*Cldn10*
213	0.963	1.9361393	0.926	1.4832462	1	0.314	Aqp4+_Astrocyte	*Atp1a2*
214	0.962	1.8368188	0.924	1.3292766	0.987	0.273	Aqp4+_Astrocyte	*S1pr1*
215	0.961	1.9149391	0.922	1.2797333	0.998	0.423	Aqp4+_Astrocyte	*Sparcl1*

216	0.958	1.8455617	0.916	1.336527	0.999	0.377	Aqp4+_Astrocyte	*Acsl3*
217	0.957	1.6643303	0.914	1.3033726	0.978	0.18	Aqp4+_Astrocyte	*Lsamp*
218	0.957	1.8522403	0.914	1.3552861	0.971	0.209	Aqp4+_Astrocyte	*Atp1b2*
219	0.957	2.0735639	0.914	1.4494618	0.961	0.158	Aqp4+_Astrocyte	*Fjx1*
220	0.955	1.8240053	0.91	1.3858078	0.973	0.136	Aqp4+_Astrocyte	*Ntm*
221	0.953	1.6558557	0.906	1.2861448	0.955	0.143	Aqp4+_Astrocyte	*Acsl6*
222	0.951	1.7970021	0.902	1.179941	0.973	0.439	Aqp4+_Astrocyte	*Tspan7*
223	0.951	1.6902786	0.902	1.130926	0.982	0.445	Aqp4+_Astrocyte	*Scg3*
224	0.951	1.680452	0.902	1.1576235	0.982	0.406	Aqp4+_Astrocyte	*Gpm6a*
225	0.95	1.825545	0.9	1.3960352	0.963	0.122	Aqp4+_Astrocyte	*Aqp4*
226	0.95	1.6529683	0.9	1.2411752	0.999	0.358	Aqp4+_Astrocyte	*Nrxn1*
227	0.949	1.8837946	0.898	1.4690057	0.991	0.226	Aqp4+_Astrocyte	*Cspg5*
228	0.949	1.5790043	0.898	1.1450928	0.998	0.415	Aqp4+_Astrocyte	*Ptprz1*
229	0.946	1.804769	0.892	1.3725794	0.915	0.059	Aqp4+_Astrocyte	*Cxcl14*
230	0.945	1.6974951	0.89	1.1295117	0.992	0.455	Aqp4+_Astrocyte	*Dclk1*
231	0.944	1.9737171	0.888	1.4729589	0.982	0.265	Aqp4+_Astrocyte	*Aldoc*
232	0.942	1.8725184	0.884	1.3864413	0.996	0.408	Aqp4+_Astrocyte	*Clu*
233	0.942	1.7158116	0.884	1.3072645	0.916	0.078	Aqp4+_Astrocyte	*Kcnk1*
234	0.94	1.5726948	0.88	1.2455864	0.978	0.198	Aqp4+_Astrocyte	*Prex2*
235	0.938	1.5046834	0.876	1.2132482	0.952	0.17	Aqp4+_Astrocyte	*Mmd2*
236	0.936	1.6217835	0.872	0.9461496	1	0.843	Aqp4+_Astrocyte	*Apoe*
237	0.933	1.7473193	0.866	1.2293241	0.943	0.259	Aqp4+_Astrocyte	*Msmo1*
238	0.932	1.4675067	0.864	1.0404107	0.982	0.459	Aqp4+_Astrocyte	*Prnp*
239	0.93	1.5160892	0.86	1.2033213	0.896	0.057	Aqp4+_Astrocyte	*Slc7a10*
240	0.926	1.464958	0.852	1.1786244	0.961	0.22	Aqp4+_Astrocyte	*Rorb*
241	0.926	1.6257179	0.852	1.2421357	0.969	0.282	Aqp4+_Astrocyte	*Mfge8*
242	0.925	1.4172508	0.85	1.0820301	0.993	0.427	Aqp4+_Astrocyte	*Ndrg2*
243	0.925	1.4729346	0.85	1.1962922	0.913	0.092	Aqp4+_Astrocyte	*Gabrb1*

244	0.924	1.3521059	0.848	1.1164552	0.892	0.078	Aqp4+_Astrocyte	*Fgfr3*
245	0.921	1.7226491	0.842	1.3073161	0.923	0.145	Aqp4+_Astrocyte	*Pla2g7*
246	0.915	1.4456547	0.83	1.1559071	0.921	0.158	Aqp4+_Astrocyte	*Ednrb*
247	0.915	1.3225797	0.83	1.1146176	0.946	0.176	Aqp4+_Astrocyte	*Sdc4*
248	0.914	1.3407852	0.828	1.1075265	0.851	0.033	Aqp4+_Astrocyte	*Gpc5*
249	0.914	1.3940158	0.828	1.141612	0.883	0.101	Aqp4+_Astrocyte	*Acsbg1*
250	0.912	1.3840809	0.824	1.003429	0.995	0.572	Aqp4+_Astrocyte	*Ptn*
251	0.893	2.4829936	0.786	1.2729485	0.895	0.421	Proliferating_Neurobl	*Hmgb2*
252	0.893	1.6183321	0.786	1.1136003	0.955	0.465	Proliferating_Neurobl	*Ccnd2*
253	0.884	1.3058167	0.768	0.5634693	0.937	0.865	Proliferating_Neurobl	*Hmgb1*
254	0.883	1.7220136	0.766	1.0451111	0.906	0.492	Proliferating_Neurobl	*Hmgn2*
255	0.879	1.8184169	0.758	0.8793174	0.917	0.704	Proliferating_Neurobl	*H2afz*
256	0.867	1.4170696	0.734	1.0001867	0.852	0.307	Proliferating_Neurobl	*Nasp*
257	0.863	1.3505207	0.726	0.8070068	0.89	0.59	Proliferating_Neurobl	*H2afv*
258	0.862	1.4248011	0.724	1.0317286	0.823	0.195	Proliferating_Neurobl	*Lmnb1*
259	0.862	1.269905	0.724	0.7919347	0.883	0.57	Proliferating_Neurobl	*Ran*
260	0.854	1.334393	0.708	0.8783098	0.866	0.436	Proliferating_Neurobl	*Anp32b*
261	0.852	2.0801932	0.704	1.3115072	0.718	0.028	Proliferating_Neurobl	*Pclaf*
262	0.851	1.5006706	0.702	1.0587609	0.77	0.131	Proliferating_Neurobl	*Dut*
263	0.849	1.3137851	0.698	0.937437	0.826	0.28	Proliferating_Neurobl	*Selenoh*
264	0.847	1.4294024	0.694	0.9939668	0.841	0.287	Proliferating_Neurobl	*Mdk*
265	0.846	1.127599	0.692	0.8192325	0.864	0.433	Proliferating_Neurobl	*Cdk4*
266	0.844	0.9220876	0.688	0.5237149	0.93	0.789	Proliferating_Neurobl	*Hnrnpa3*
267	0.843	1.0981949	0.686	0.7353424	0.882	0.544	Proliferating_Neurobl	*Nap1l1*
268	0.842	1.1414099	0.684	0.8102477	0.866	0.418	Proliferating_Neurobl	*Tmpo*
269	0.839	1.1592031	0.678	0.7783523	0.87	0.493	Proliferating_Neurobl	*Ranbp1*
270	0.835	1.1171137	0.67	0.8979288	0.867	0.304	Proliferating_Neurobl	*Hmgb3*
271	0.835	0.9751779	0.67	0.6279421	0.898	0.662	Proliferating_Neurobl	*Hmgn1*

272	0.833	1.2248176	0.666	0.7816934	0.874	0.497	Proliferating_Neurobl	*Dek*
273	0.833	0.8967236	0.666	0.6064921	0.916	0.659	Proliferating_Neurobl	*Cbx3*
274	0.832	1.4537727	0.664	1.0351749	0.679	0.024	Proliferating_Neurobl	*Rrm2*
275	0.831	1.1374141	0.662	0.8749051	0.809	0.235	Proliferating_Neurobl	*Ezh2*
276	0.829	2.2542933	0.658	1.2919651	0.672	0.024	Proliferating_Neurobl	*Top2a*
277	0.826	0.9926832	0.652	0.8147298	0.771	0.161	Proliferating_Neurobl	*Mcm7*
278	0.825	0.9247692	0.65	0.5444316	0.901	0.731	Proliferating_Neurobl	*Npm1*
279	0.825	0.9478758	0.65	0.6009729	0.901	0.674	Proliferating_Neurobl	*Set*
280	0.824	1.0618881	0.648	0.9876199	0.951	0.358	Proliferating_Neurobl	*Sox11*
281	0.824	1.2351523	0.648	0.9121343	0.708	0.074	Proliferating_Neurobl	*Lockd*
282	0.821	1.0092305	0.642	0.7192434	0.864	0.464	Proliferating_Neurobl	*Hdgf*
283	0.82	0.8738548	0.64	0.5218663	0.903	0.744	Proliferating_Neurobl	*Sumo2*
284	0.82	1.0010397	0.64	0.702739	0.885	0.552	Proliferating_Neurobl	*Hnrnpab*
285	0.819	0.9819091	0.638	0.694797	0.859	0.499	Proliferating_Neurobl	*Rbm3*
286	0.814	0.8635046	0.628	0.657779	0.887	0.517	Proliferating_Neurobl	*Snrpf*
287	0.813	1.2731316	0.626	0.9138115	0.721	0.149	Proliferating_Neurobl	*Smc2*
288	0.813	1.0722539	0.626	0.8424372	0.812	0.236	Proliferating_Neurobl	*Cdca7*
289	0.812	0.9580724	0.624	0.7786148	0.664	0.047	Proliferating_Neurobl	*Cks1b*
290	0.81	0.8500873	0.62	0.6071766	0.897	0.623	Proliferating_Neurobl	*Snrpg*
291	0.81	0.8324334	0.62	0.6433244	0.9	0.546	Proliferating_Neurobl	*Psip1*
292	0.81	1.0031479	0.62	0.7277049	0.837	0.406	Proliferating_Neurobl	*Anp32e*
293	0.806	0.8870474	0.612	0.7314976	0.755	0.202	Proliferating_Neurobl	*Dnajc9*
294	0.806	1.2527208	0.612	0.869092	0.739	0.184	Proliferating_Neurobl	*Smc4*
295	0.804	1.1608874	0.608	0.8772623	0.625	0.023	Proliferating_Neurobl	*Hells*
296	0.803	1.1589771	0.606	0.6184335	0.876	0.686	Proliferating_Neurobl	*Tuba1b*
297	0.803	1.0715021	0.606	0.8280895	0.665	0.079	Proliferating_Neurobl	*Mcm6*
298	0.8	0.9395633	0.6	0.6921639	0.823	0.402	Proliferating_Neurobl	*Cdk2ap1*
299	0.8	0.8607203	0.6	0.701667	0.841	0.333	Proliferating_Neurobl	*Nsd2*

300	0.799	0.9015869	0.598	0.6840107	0.905	0.602	Proliferating_Neurobl	*Hnrnpa1*
301	1	4.4120474	1	1.6847835	1	0.733	Oligodendrocyte	*Plp1*
302	1	3.5628063	1	2.031192	1	0.181	Oligodendrocyte	*Mbp*
303	0.997	3.2067608	0.994	1.9305556	0.995	0.138	Oligodendrocyte	*Mag*
304	0.995	3.2372918	0.99	1.9615472	0.992	0.116	Oligodendrocyte	*Mobp*
305	0.994	2.5815878	0.988	1.6839527	0.994	0.172	Oligodendrocyte	*Ptprd*
306	0.992	3.6932748	0.984	1.8816766	0.992	0.328	Oligodendrocyte	*Mal*
307	0.99	2.6925579	0.98	1.5011412	0.99	0.438	Oligodendrocyte	*Aplp1*
308	0.987	2.5249756	0.974	1.6884043	0.982	0.136	Oligodendrocyte	*Enpp2*
309	0.986	3.1884029	0.972	1.7104824	0.984	0.372	Oligodendrocyte	*Cnp*
310	0.986	2.3461644	0.972	1.6043807	0.985	0.164	Oligodendrocyte	*Ttll7*
311	0.986	3.2307477	0.972	1.9379559	0.978	0.125	Oligodendrocyte	*Cldn11*
312	0.983	2.2679637	0.966	1.6260484	0.97	0.021	Oligodendrocyte	*Gjc3*
313	0.983	2.9552307	0.966	1.8562247	0.971	0.087	Oligodendrocyte	*Tspan2*
314	0.981	2.4946999	0.962	1.6675641	0.983	0.186	Oligodendrocyte	*Sept4*
315	0.98	1.9956467	0.96	1.4802411	0.97	0.072	Oligodendrocyte	*Map7*
316	0.98	2.4340805	0.96	1.693945	0.965	0.045	Oligodendrocyte	*Ugt8a*
317	0.979	1.4765193	0.958	0.7569078	0.998	0.806	Oligodendrocyte	*Qk*
318	0.977	2.543427	0.954	1.4934873	0.986	0.345	Oligodendrocyte	*Trf*
319	0.977	2.8381107	0.954	1.8321945	0.96	0.07	Oligodendrocyte	*Ermn*
320	0.976	2.7458521	0.952	1.7965817	0.958	0.061	Oligodendrocyte	*Mog*
321	0.973	1.6984625	0.946	0.9722023	0.986	0.658	Oligodendrocyte	*Kif1b*
322	0.973	1.8618794	0.946	1.4042875	0.961	0.098	Oligodendrocyte	*Abca2*
323	0.971	1.8878373	0.942	1.0532621	0.992	0.64	Oligodendrocyte	*Scd2*
324	0.971	1.9260602	0.942	1.4380459	0.963	0.105	Oligodendrocyte	*Edil3*
325	0.971	1.7983819	0.942	1.3977544	0.953	0.017	Oligodendrocyte	*Slc24a2*
326	0.969	2.1873606	0.938	1.5713625	0.948	0.054	Oligodendrocyte	*Olig1*
327	0.969	2.1063998	0.938	1.5220783	0.946	0.026	Oligodendrocyte	*Kcna1*

328	0.969	2.6175084	0.938	1.5886453	0.968	0.236	Oligodendrocyte	*Car2*
329	0.968	2.924212	0.936	1.7324956	0.96	0.214	Oligodendrocyte	*Cryab*
330	0.967	2.2734887	0.934	1.4849538	0.957	0.243	Oligodendrocyte	*Dbndd2*
331	0.966	1.8951951	0.932	1.4116042	0.977	0.145	Oligodendrocyte	*Ank3*
332	0.966	1.7005944	0.932	1.2984759	0.962	0.138	Oligodendrocyte	*Phldb1*
333	0.966	1.8532423	0.932	1.4297154	0.936	0.02	Oligodendrocyte	*Fa2h*
334	0.966	1.8034784	0.932	1.3876132	0.942	0.026	Oligodendrocyte	*Tmeff2*
335	0.963	1.6919819	0.926	1.3189039	0.946	0.07	Oligodendrocyte	*Aatk*
336	0.963	1.7850565	0.926	1.3883992	0.934	0.032	Oligodendrocyte	*Aspa*
337	0.962	1.8543764	0.924	1.4057369	0.945	0.07	Oligodendrocyte	*Ppp1r16b*
338	0.962	1.7939011	0.924	1.3859842	0.934	0.039	Oligodendrocyte	*Cntn2*
339	0.96	1.7953915	0.92	1.3018988	0.958	0.207	Oligodendrocyte	*Ccp110*
340	0.959	1.7842958	0.918	1.0906751	0.969	0.521	Oligodendrocyte	*Syt11*
341	0.957	1.5967983	0.914	1.1513402	0.982	0.362	Oligodendrocyte	*Mapt*
342	0.957	1.8412398	0.914	1.3379622	0.938	0.079	Oligodendrocyte	*Nfasc*
343	0.956	1.7056619	0.912	1.3207433	0.949	0.125	Oligodendrocyte	*Epb41l3*
344	0.956	1.8168547	0.912	1.3841225	0.926	0.068	Oligodendrocyte	*Gpr37*
345	0.956	1.6595684	0.912	1.2754526	0.92	0.019	Oligodendrocyte	*1700047M11Rik*
346	0.954	1.7060836	0.908	1.2667365	0.963	0.273	Oligodendrocyte	*Slc12a2*
347	0.954	2.3990728	0.908	1.6136776	0.939	0.149	Oligodendrocyte	*Apod*
348	0.953	1.8809908	0.906	1.3348431	0.969	0.275	Oligodendrocyte	*Gatm*
349	0.952	2.3911753	0.904	1.5248366	0.934	0.193	Oligodendrocyte	*Tubb4a*
350	0.952	1.9445053	0.904	1.3909075	0.93	0.165	Oligodendrocyte	*Desi1*
351	0.994	2.7432174	0.988	1.2217304	1	0.742	Ependymal_Cell	*Dbi*
352	0.978	1.9274237	0.956	0.8053439	0.996	0.872	Ependymal_Cell	*Hsp90aa1*
353	0.973	2.5385406	0.946	1.3744461	0.969	0.498	Ependymal_Cell	*Tubb4b*
354	0.964	3.1354573	0.928	1.8139699	0.942	0.187	Ependymal_Cell	*Rarres2*
355	0.963	3.0379986	0.926	1.8907262	0.931	0.054	Ependymal_Cell	*Ccdc153*

356	0.962	2.5220472	0.924	1.5450083	0.947	0.269	Ependymal_Cell	*Chchd10*
357	0.959	2.0586389	0.918	1.3401767	0.986	0.466	Ependymal_Cell	*Mt3*
358	0.95	2.9407267	0.9	1.8366173	0.906	0.053	Ependymal_Cell	*Tmem212*
359	0.949	2.6456706	0.898	1.7045986	0.909	0.082	Ependymal_Cell	*1700094D03Rik*
360	0.943	1.8599721	0.886	1.1119476	0.978	0.528	Ependymal_Cell	*Nnat*
361	0.934	2.41631	0.868	1.6200158	0.874	0.027	Ependymal_Cell	*Fam183b*
362	0.933	2.4889607	0.866	1.6475665	0.874	0.037	Ependymal_Cell	*Dynlrb2*
363	0.922	2.3838509	0.844	1.5802606	0.852	0.042	Ependymal_Cell	*Rsph1*
364	0.922	2.0045669	0.844	1.2876428	0.899	0.305	Ependymal_Cell	*Anxa5*
365	0.916	1.2107383	0.832	0.5623541	0.974	0.878	Ependymal_Cell	*Calm1*
366	0.913	2.3108181	0.826	1.5463933	0.833	0.031	Ependymal_Cell	*1110017D15Rik*
367	0.912	2.3479529	0.824	1.5457234	0.838	0.064	Ependymal_Cell	*Gm19935*
368	0.91	2.1907358	0.82	1.4252243	0.847	0.151	Ependymal_Cell	*Ascc1*
369	0.91	1.5268147	0.82	0.9703618	0.993	0.687	Ependymal_Cell	*Mt1*
370	0.91	2.6301127	0.82	1.6250382	0.835	0.064	Ependymal_Cell	*Mia*
371	0.905	2.0887198	0.81	1.4322101	0.82	0.032	Ependymal_Cell	*Cfap126*
372	0.904	2.196772	0.808	1.4816102	0.817	0.032	Ependymal_Cell	*Meig1*
373	0.893	1.7024385	0.786	1.0728375	0.871	0.404	Ependymal_Cell	*Nudt4*
374	0.89	1.9426135	0.78	1.1954018	0.846	0.354	Ependymal_Cell	*Elof1*
375	0.89	1.5571245	0.78	1.0610139	0.915	0.395	Ependymal_Cell	*Vim*
376	0.889	2.0963118	0.778	1.4202859	0.784	0.021	Ependymal_Cell	*Ak7*
377	0.888	1.8018415	0.776	1.2360423	0.817	0.156	Ependymal_Cell	*Bphl*
378	0.886	1.904747	0.772	1.3342652	0.787	0.051	Ependymal_Cell	*Foxj1*
379	0.886	1.9652792	0.772	1.352734	0.779	0.017	Ependymal_Cell	*1700012B09Rik*
380	0.884	1.8778848	0.768	1.2808082	0.8	0.111	Ependymal_Cell	*Mns1*
381	0.884	1.8942299	0.768	1.3156821	0.781	0.041	Ependymal_Cell	*Mlf1*
382	0.882	1.8952554	0.764	1.3284509	0.774	0.026	Ependymal_Cell	*1700007K13Rik*
383	0.88	1.569437	0.76	0.9262103	0.861	0.533	Ependymal_Cell	*Nudc*

384	0.878	1.8756703	0.756	1.2977965	0.78	0.062	Ependymal_Cell	*Enkur*
385	0.876	1.8181918	0.752	1.2467068	0.796	0.129	Ependymal_Cell	*Tppp3*
386	0.872	1.6000115	0.744	1.2604974	0.843	0.138	Ependymal_Cell	*Tm4sf1*
387	0.868	1.7257728	0.736	1.2330022	0.745	0.019	Ependymal_Cell	*Odf3b*
388	0.867	1.6591306	0.734	1.186661	0.743	0.02	Ependymal_Cell	*Capsl*
389	0.864	1.7958268	0.728	1.2404573	0.734	0.012	Ependymal_Cell	*Sntn*
390	0.864	2.0264151	0.728	1.3600707	0.744	0.047	Ependymal_Cell	*Aebp1*
391	0.864	1.7076277	0.728	1.2004086	0.736	0.015	Ependymal_Cell	*Ccdc113*
392	0.861	1.6660925	0.722	1.1852138	0.728	0.012	Ependymal_Cell	*1700016K19Rik*
393	0.86	1.6405867	0.72	1.1936962	0.845	0.213	Ependymal_Cell	*Ifitm3*
394	0.858	1.5756884	0.716	1.0953497	0.804	0.231	Ependymal_Cell	*Hspa2*
395	0.858	1.660053	0.716	1.1847136	0.722	0.015	Ependymal_Cell	*1700001C02Rik*
396	0.855	1.3107858	0.71	0.791412	0.853	0.56	Ependymal_Cell	*Txn1*
397	0.855	1.6062819	0.71	1.1446771	0.718	0.014	Ependymal_Cell	*Hdc*
398	0.854	1.5610064	0.708	1.0594491	0.809	0.245	Ependymal_Cell	*Csrp2*
399	0.854	1.3544741	0.708	0.9610689	0.894	0.426	Ependymal_Cell	*Gstm1*
400	0.854	1.7931174	0.708	1.2203038	0.744	0.086	Ependymal_Cell	*Riiad1*
401	0.912	2.4771214	0.824	1.6651209	0.876	0.122	Pericyte	*Igfbp7*
402	0.818	4.4457013	0.636	1.9650427	0.65	0.036	Pericyte	*Vtn*
403	0.817	2.0777935	0.634	1.287156	0.678	0.091	Pericyte	*Itga1*
404	0.805	3.5889954	0.61	1.7695648	0.624	0.035	Pericyte	*Rgs5*
405	0.795	1.5936504	0.59	1.0568819	0.696	0.183	Pericyte	*Gng11*
406	0.788	1.5073491	0.576	0.7804734	0.844	0.604	Pericyte	*Ptn*
407	0.788	1.3758046	0.576	1.0956386	0.707	0.14	Pericyte	*Hspb1*
408	0.786	1.4182548	0.572	1.0003694	0.705	0.202	Pericyte	*Epas1*
409	0.786	2.3254437	0.572	1.3446619	0.575	0.009	Pericyte	*Ndufa4l2*
410	0.785	1.7916269	0.57	1.1233799	0.641	0.128	Pericyte	*Col4a1*
411	0.781	2.3208848	0.562	1.3224756	0.565	0.009	Pericyte	*Higd1b*
412	0.781	1.0525018	0.562	0.7116558	0.84	0.556	Pericyte	*Sptbn1*
413	0.779	1.4636295	0.558	1.0242941	0.679	0.152	Pericyte	*Tm4sf1*
414	0.778	1.4300344	0.556	0.9763076	0.689	0.217	Pericyte	*Utrn*
415	0.778	2.4811834	0.556	1.185993	0.681	0.366	Pericyte	*Cald1*
416	0.777	1.5394819	0.554	1.0295383	0.616	0.095	Pericyte	*Arhgap29*
417	0.775	2.2698283	0.55	1.2610983	0.595	0.104	Pericyte	*Pdgfrb*
418	0.773	2.4613945	0.546	1.3286431	0.581	0.077	Pericyte	*Myl9*
419	0.77	2.2854099	0.54	1.291892	0.544	0.009	Pericyte	*Atp13a5*
420	0.77	2.0271813	0.54	1.1958159	0.541	0.005	Pericyte	*Slc6a20a*
421	0.766	1.476864	0.532	0.9833818	0.603	0.108	Pericyte	*Col4a2*
422	0.766	0.865996	0.532	0.7912063	0.855	0.464	Pericyte	*Sparcl1*

423	0.766	2.3304572	0.532	1.2975199	0.55	0.035	Pericyte	*Rgs4*
424	0.764	2.2381921	0.528	1.2425385	0.539	0.024	Pericyte	*Sod3*
425	0.764	1.6725241	0.528	1.050208	0.563	0.063	Pericyte	*Cp*
426	0.763	1.7440183	0.526	1.0757581	0.561	0.073	Pericyte	*Nbl1*
427	0.762	1.6665492	0.524	1.1059528	0.592	0.074	Pericyte	*Car4*
428	0.76	1.6522317	0.52	1.0021015	0.602	0.176	Pericyte	*Myo1b*
429	0.759	1.7118545	0.518	1.0649876	0.534	0.031	Pericyte	*Nid1*
430	0.759	1.4596219	0.518	0.9442913	0.599	0.146	Pericyte	*Rbpms*
431	0.758	2.1385896	0.516	1.2182799	0.532	0.03	Pericyte	*P2ry14*
432	0.756	1.5391204	0.512	0.9592516	0.584	0.132	Pericyte	*Dlc1*
433	0.755	1.604732	0.51	1.0058648	0.556	0.071	Pericyte	*Ebf1*
434	0.752	1.7747311	0.504	0.9716736	0.682	0.37	Pericyte	*Atp1a2*
435	0.751	2.0237026	0.502	1.1436587	0.508	0.01	Pericyte	*Ifitm1*
436	0.75	1.5781379	0.5	0.9769584	0.557	0.1	Pericyte	*Gjc1*
437	0.75	2.2072471	0.5	1.2049917	0.504	0.004	Pericyte	*Kcnj8*
438	0.749	1.2851489	0.498	0.9581495	0.643	0.213	Pericyte	*Sept4*
439	0.749	1.799547	0.498	1.0825438	0.523	0.047	Pericyte	*Gucy1b1*
440	0.748	1.2120218	0.496	0.668963	0.712	0.526	Pericyte	*Itgb1*
441	0.747	2.070553	0.494	1.1661385	0.496	0.005	Pericyte	*Abcc9*
442	0.746	1.6216603	0.492	1.0185606	0.501	0.017	Pericyte	*Colec12*
443	0.746	1.625566	0.492	1.0127128	0.502	0.013	Pericyte	*Zic1*
444	0.746	1.6488831	0.492	1.0063635	0.529	0.073	Pericyte	*Lamc1*
445	0.745	0.7564169	0.49	0.6909805	0.932	0.542	Pericyte	*Sparc*
446	0.745	1.5955053	0.49	0.9854899	0.515	0.047	Pericyte	*Slc19a1*
447	0.743	1.4985791	0.486	0.9493236	0.513	0.049	Pericyte	*Phldb2*
448	0.742	1.6738072	0.484	1.0237875	0.488	0.007	Pericyte	*Cspg4*
449	0.741	1.1166453	0.482	0.8265898	0.677	0.227	Pericyte	*Ifitm3*
450	0.739	1.6143137	0.478	0.9993342	0.487	0.016	Pericyte	*Notch3*
451	0.972	3.2970268	0.944	1.9692326	0.948	0.059	GABAergic_Neuron	*Arpp21*
452	0.968	2.7051157	0.936	1.4352286	0.959	0.498	GABAergic_Neuron	*Ppp3ca*
453	0.963	2.7292805	0.926	1.7013046	0.938	0.059	GABAergic_Neuron	*Syt1*
454	0.96	2.2634184	0.92	1.5916289	0.928	0.048	GABAergic_Neuron	*Gnal*
455	0.958	2.5872041	0.916	1.7142839	0.918	0.01	GABAergic_Neuron	*Gpr88*
456	0.953	2.7300982	0.906	1.7972663	0.907	0.008	GABAergic_Neuron	*Grm5*
457	0.95	2.7217223	0.9	1.4251808	0.938	0.488	GABAergic_Neuron	*Atp2b1*
458	0.948	3.3795557	0.896	1.9320789	0.907	0.102	GABAergic_Neuron	*Pde10a*
459	0.945	2.5589884	0.89	1.6413653	0.907	0.132	GABAergic_Neuron	*Atp2b2*
460	0.943	2.5076547	0.886	1.6357241	0.897	0.079	GABAergic_Neuron	*Ndrg4*
461	0.942	2.5774845	0.884	1.6794559	0.897	0.099	GABAergic_Neuron	*Grin2b*
462	0.941	2.3505865	0.882	1.5692652	0.897	0.109	GABAergic_Neuron	*Baiap2*
463	0.941	2.4117598	0.882	1.6306793	0.887	0.023	GABAergic_Neuron	*Camk2b*

464	0.93	3.5523749	0.86	1.8302546	0.876	0.074	GABAergic_Neuron	*Pcp4*
465	0.928	2.1616828	0.856	1.49222	0.866	0.037	GABAergic_Neuron	*Plppr4*
466	0.927	2.0884407	0.854	1.4091113	0.876	0.082	GABAergic_Neuron	*Ppp1r1b*
467	0.927	1.8451181	0.854	1.3664463	0.856	0.006	GABAergic_Neuron	*Rasgef1a*
468	0.927	1.7169818	0.854	0.9991379	0.938	0.551	GABAergic_Neuron	*Atp2a2*
469	0.924	2.3570871	0.848	1.4259133	0.887	0.303	GABAergic_Neuron	*Phactr1*
470	0.924	2.4078404	0.848	1.6100576	0.856	0.038	GABAergic_Neuron	*Rgs9*
471	0.924	2.3294653	0.848	1.5122376	0.866	0.113	GABAergic_Neuron	*Prkcb*
472	0.922	2.07302	0.844	1.4194025	0.866	0.079	GABAergic_Neuron	*Rgs7bp*
473	0.92	1.9980724	0.84	1.4355429	0.845	0.015	GABAergic_Neuron	*Cpne5*
474	0.92	2.0674955	0.84	1.4717135	0.845	0.022	GABAergic_Neuron	*Ptpn5*
475	0.92	3.6034439	0.84	2.0014502	0.845	0.034	GABAergic_Neuron	*Snhg11*
476	0.92	2.0328755	0.84	1.3861497	0.866	0.142	GABAergic_Neuron	*Chn1*
477	0.92	2.1140002	0.84	1.472422	0.845	0.018	GABAergic_Neuron	*Scn2a*
478	0.918	2.243044	0.836	1.5305088	0.845	0.048	GABAergic_Neuron	*Adcy5*
479	0.916	2.0599447	0.832	1.4496898	0.835	0.01	GABAergic_Neuron	*Camkv*
480	0.915	1.8488497	0.83	1.345873	0.835	0.018	GABAergic_Neuron	*Tmem158*
481	0.915	1.7913782	0.83	1.323575	0.835	0.012	GABAergic_Neuron	*Grin1*
482	0.914	2.3038031	0.828	1.4560291	0.835	0.02	GABAergic_Neuron	*Nrgn*
483	0.913	1.66173	0.826	1.1631664	0.938	0.409	GABAergic_Neuron	*Gria2*
484	0.912	1.7678545	0.824	1.2969941	0.845	0.074	GABAergic_Neuron	*Kcnb1*
485	0.912	2.0623121	0.824	1.4095871	0.835	0.043	GABAergic_Neuron	*Ptprn*
486	0.91	1.7970238	0.82	1.2742974	0.856	0.144	GABAergic_Neuron	*Cplx2*
487	0.908	1.9861638	0.816	1.3835149	0.825	0.027	GABAergic_Neuron	*Atp1a3*
488	0.905	1.8017318	0.81	1.3173001	0.825	0.046	GABAergic_Neuron	*Cacna2d3*
489	0.904	1.6870327	0.808	1.2345943	0.814	0.016	GABAergic_Neuron	*Camk2a*
490	0.904	1.9550206	0.808	1.3434692	0.845	0.116	GABAergic_Neuron	*Itpr1*
491	0.904	1.9427789	0.808	1.3817809	0.825	0.053	GABAergic_Neuron	*Pcsk2*

492	0.902	1.908813	0.804	1.3446162	0.825	0.078	GABAergic_Neuron	*Pde1b*
493	0.901	1.8234696	0.802	1.3250044	0.804	0.004	GABAergic_Neuron	*Trank1*
494	0.901	1.7188453	0.802	1.2623386	0.814	0.032	GABAergic_Neuron	*Spock3*
495	0.9	1.8565824	0.8	1.3312025	0.804	0.009	GABAergic_Neuron	*Gria3*
496	0.9	1.7287923	0.8	1.2649006	0.814	0.03	GABAergic_Neuron	*Erc2*
497	0.9	1.9673247	0.8	1.3065631	0.845	0.171	GABAergic_Neuron	*Dgkb*
498	0.899	1.6368749	0.798	1.1990034	0.825	0.066	GABAergic_Neuron	*Cit*
499	0.898	2.3749307	0.796	1.3925929	0.876	0.373	GABAergic_Neuron	*Meg3*
500	0.898	1.9472063	0.796	1.2029237	0.876	0.322	GABAergic_Neuron	*Camk2n1*
501	0.995	2.855675	0.99	1.9161898	0.991	0.003	Erythrocyt	*Alas2*
502	0.995	6.3235758	0.99	2.8097296	0.991	0.163	Erythrocyt	*Hba-a2*
503	0.995	6.3296537	0.99	2.8074568	0.991	0.183	Erythrocyt	*Hba-a1*
504	0.995	6.6116801	0.99	2.839841	0.991	0.073	Erythrocyt	*Hbb-bt*
505	0.994	6.5608539	0.988	2.6473007	0.991	0.371	Erythrocyt	*Hbb-bs*
506	0.985	2.1773191	0.97	1.5913672	0.974	0.023	Erythrocyt	*Snca*
507	0.979	1.8110097	0.958	1.4118915	0.966	0.049	Erythrocyt	*Ube2l6*
508	0.956	1.8297309	0.912	1.1853996	0.974	0.412	Erythrocyt	*Mkrn1*
509	0.952	1.4437411	0.904	1.1826669	0.914	0.018	Erythrocyt	*Isg20*
510	0.949	1.6365338	0.898	1.2728802	0.94	0.086	Erythrocyt	*Tent5c*
511	0.947	2.32148	0.894	1.4841624	0.931	0.225	Erythrocyt	*Bpgm*
512	0.908	1.137383	0.816	0.9498233	0.897	0.16	Erythrocyt	*Fech*
513	0.899	1.2210575	0.798	0.990391	0.879	0.127	Erythrocyt	*Slc25a37*
514	0.886	1.0916764	0.772	0.9176894	0.888	0.205	Erythrocyt	*Epb41*
515	0.869	0.9833547	0.738	0.8178397	0.879	0.256	Erythrocyt	*Slc25a39*
516	0.853	0.7956581	0.706	0.7285838	0.888	0.281	Erythrocyt	*March2*
517	0.837	0.8350385	0.674	0.7411477	0.69	0.015	Erythrocyt	*Gda*
518	0.825	0.7561783	0.65	0.6895021	0.776	0.152	Erythrocyt	*Isca1*
519	0.808	1.1308438	0.616	0.8284598	0.655	0.037	Erythrocyt	*Rsad2*
520	0.804	0.8387608	0.608	0.5721473	0.966	0.65	Erythrocyt	*Sec61g*
521	0.796	1.4468067	0.592	0.8226764	0.845	0.455	Erythrocyt	*Gpx1*
522	0.793	0.7115576	0.586	0.5272135	0.922	0.638	Erythrocyt	*Bnip3l*
523	0.774	0.5569544	0.548	0.5391255	0.75	0.218	Erythrocyt	*Ccndbp1*
524	0.773	0.6263815	0.546	0.5794202	0.672	0.141	Erythrocyt	*Ube2o*
525	0.765	0.6232655	0.53	0.5617321	0.56	0.035	Erythrocyt	*Cdr2*
526	0.727	0.6371237	0.454	0.5279777	0.457	0.002	Erythrocyt	*Rec114*
527	0.716	0.6867343	0.432	0.5324398	0.431	0	Erythrocyt	*Gypa*
